# Natural deep eutectic solvents (NaDES): green solvents for pharmaceutical applications and beyond†

**DOI:** 10.1039/d4gc06386d

**Published:** 2025-04-28

**Authors:** Emma Chevé-Kools, Young Hae Choi, Catherine Roullier, Gwenaël Ruprich-Robert, Raphaël Grougnet, Florence Chapeland-Leclerc, Frank Hollmann

**Affiliations:** a Cibles Thérapeutiques et Conception de Médicaments (CiTCoM, UMR 8038 CNRS), Faculté de Pharmacie de Paris, Université Paris Cité Paris France emma.cheve@free.fr florence.leclerc@u-paris.fr; b Natural Products Laboratory, Institute of Biology, Leiden University Leiden The Netherlands; c Institut des Substances et Organismes de la Mer (ISOMER, UR 2160), Faculté de Pharmacie de Nantes, Nantes Université France; d Department of Biotechnology, Delft University of Technology Delft The Netherlands

## Abstract

Composed of various biosourced metabolites, NaDES offer significant economic, health, and environmental benefits. Their remarkable ability to interact with target compounds through non-covalent bonds enhances their versatility. As solvents, excipients, cofactors, catalysts, solubilisation promoters, stabilisers, and absorption agents, NaDES provide distinct advantages over conventional substances and can even act as active compounds themselves. Furthermore, their role in advancing innovative synthesis and formulation strategies, particularly in nanotechnology and biotechnology, is driving research in these areas. This review is the first to explore all the potential applications of NaDES in the pharmaceutical industry, while taking a comprehensive look at the theory behind them. It gives a precise definition of NaDES and describes their composition, characteristics, molecular interactions, preparation, stability and recovery. It presents detailed applications in pharmaceutical synthesis, extraction and formulation, and discusses roles as active compounds or tools for innovation. Using green metrics, the efficiency of routes including NaDES is compared to that of conventional processes. Lastly, this review addresses often overlooked points such as toxicity and process limitations.

## Abstract 2

This review first provides a comprehensive overview of NaDES theory and subsequently explores all potential applications of NaDES in the pharmaceutical industry, highlighting overlooked issues such as toxicity and process limitations.

## Graphical abstract

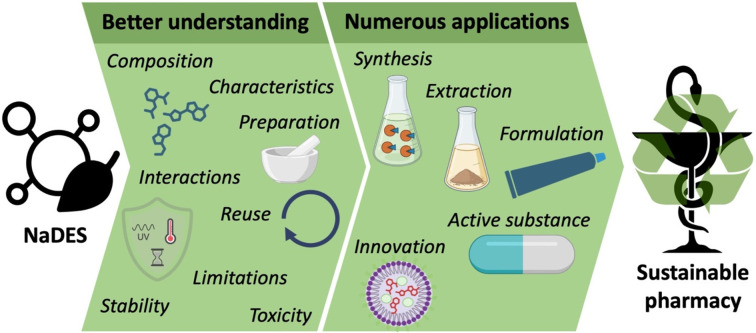

**Green foundation**

1. The review highlights natural deep eutectic solvents (NaDES) as a significant advance in green chemistry, especially for pharmaceutical applications. Their versatility is shown in roles ranging from solvents to active pharmaceutical ingredients, aiding in innovative synthesis and formulation strategies, particularly in biotechnology and nanotechnology.

2. The study of NaDES is of wider interest due to its potential to replace harmful organic solvents with safer, more sustainable alternatives. This is particularly crucial in the pharmaceutical industry, where solvent safety and environmental impact are major concerns.

3. The future of green chemistry involving NaDES looks promising as research continues to explore their broad applications, from drug synthesis to extraction and formulation. Insights from this review helps in designing greener synthesis pathways and formulations, potentially leading to wider adoption in the industry.

## Introduction

Current environmental and health concerns are compelling a reassessment of the pharmaceutical industry, with the objective of minimising the environmental impact of drug production processes. Identifying strategies that address multiple aspects of the production chain is therefore of significant interest. Natural deep eutectic solvents (NaDES), owing to their versatile properties and relatively low toxicity, present a promising solution to these challenges.

In recent years, there has been increasing interest in deep eutectic solvents (DES) across a wide range of disciplines, including chemistry, environmental science, physics and astronomy, energy, biological sciences, and pharmaceutical sciences.^1^ Within the pharmaceutical field, research on DES has predominantly focused on formulation and drug delivery systems,^1–8^ with particular attention to specific administration routes, such as topical,^9^ transdermal,^10^ ocular,^11^ and oral delivery.^12,13^ The potential for certain active pharmaceutical ingredients (APIs) to function as DES in mixtures is also being explored,^14,15^ particularly in specialised applications such as antimicrobial and anticancer therapies.^16,17^

Most reviews on NaDES focus on their applications in extraction and formulation.^7,18–20^ However, broader discussions encompassing their full potential in the pharmaceutical industry, including their role in sustainable process development and drug synthesis, remain limited. To the best of our knowledge, no comprehensive review currently provides an overarching perspective on the full range of potential applications of NaDES within the pharmaceutical industry. Moreover, a review encompassing all theoretical aspects of NaDES remains absent. With this work, we aim to encourage both researchers and industry professionals to adopt NaDES by addressing all relevant considerations, including existing limitations and challenges in the field. In particular, we highlight often-overlooked aspects such as stability, environmental impact, toxicity, and process constraints.

Wherever feasible, we have calculated green metrics to compare NaDES-based methodologies with conventional approaches. Specifically, we employed atom economy (AE), reaction mass efficiency (RME), and process mass intensity (PMI). Additional process-related data that could not be incorporated into these metrics were discussed separately. Green metrics were determined in accordance with the guidelines set by the American Chemical Society (ACS), as detailed in the ESI.†

AE is derived from the molar masses of the reactants and the product (eqn (S1)†), assuming exact stoichiometric proportions and complete reaction yields.^21^ RME refines AE by considering reaction yield and the use of excess reagents (eqn (S2)†), while excluding reagents, catalysts, and solvents. PMI, also a mass-based metric, accounts for the total mass of solvents, catalysts, and reagents (eqn (S3)†). A lower PMI indicates a greener process, with a value of one signifying a fully efficient system in which no waste is generated and all input materials are incorporated into the final product. The ACS Green Chemistry Institute Pharmaceutical Roundtable (ACS GCI PR) has designated PMI as the principal indicator of process inefficiency, economic viability, and environmental impact. Furthermore, ACS GCI PR has developed a PMI calculator to facilitate the rapid evaluation of this metric within industry, thereby fostering wider adoption and standardising impact assessment across the pharmaceutical sector.^21^

The review begins with the theory of NaDES, starting with a precise definition, followed by a description of their different compositions, physico-chemical characteristics, and chemical interactions within NaDES. Subsequently, NaDES preparation, stability and finally recovery and reuse are discussed. The second part deals with potential applications of NaDES in the pharmaceutical field. Owing to their versatile physicochemical properties, NaDES hold considerable promise across multiple pharmaceutical applications, including drug synthesis, bioactive compound extraction, formulation, active pharmaceutical ingredient (API) development, and medicinal innovation. In drug synthesis, NaDES primarily serve as reaction media, catalysts, or reagents. In extraction processes, they function as pre-treatment solvents and as polar or apolar solvents. Their role in pharmaceutical formulation is particularly valuable, as they enhance solubility, stabilisation, and bioavailability. Additionally, NaDES can act as therapeutic deep eutectic solvents (TheDES) by forming a eutectic mixture with an API or, in some cases, serving as API themselves. Lastly, they represent promising tools for innovation, particularly within the fields of biocatalysis and nanocatalysis. In the final section, the limitations and toxicity of NaDES are addressed.

## Theory of NaDES

### Definition of a deep eutectic solvent

**Fig. 1:**
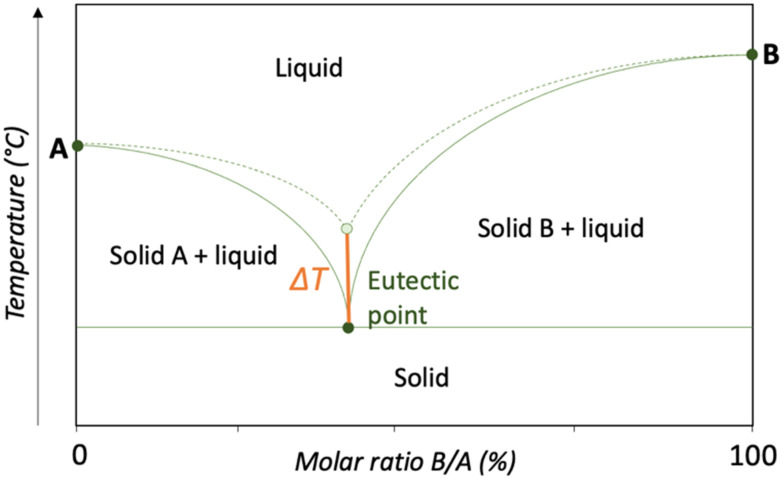
Eutectic phase diagram of the components A and B. The dashed curve illustrates the theoretical liquidus, while the solid curve depicts the experimentally determined liquidus. Dots indicate melting points and Δ*T* denotes the temperature difference between the experimental and theoretical eutectic points.

In the context of phase behaviour, the term ‘eutectic’, derived from the Greek eutektos meaning ‘easy melting’,^22^ refers to a specific type of mixture that exhibits a melting point lower than that of any other composition of the same constituents.^23^ The relationship between composition and melting temperature is visualised using a phase diagram (Fig. 1). This diagram typically illustrates the liquidus and solidus curves, which depict the boundaries between different phases as a function of the molar ratio of B to A.^1^ The liquidus curve defines the temperature above which the mixture is completely liquid, while the solidus curve (often referred to as the eutectic invariant) represents the temperature below which the mixture exists in a fully solid state. The phase diagram highlights three key melting points: the melting point of pure component A, the melting point of pure component B and the melting point at the eutectic composition, where a specific ratio of A and B results in the lowest possible melting temperature. At this eutectic point, the system forms a homogeneous liquid phase at a significantly reduced temperature. In contrast, mixtures with compositions outside of this specific eutectic ratio exhibit incomplete melting between the solidus and liquidus curves, resulting in a two-phase liquid/solid system.^1^

The liquidus curve of a given mixture can be derived using the Schröder–van Laar equation (eqn (1)), provided the melting temperature and enthalpy of each component in the mixture are known.^1^1
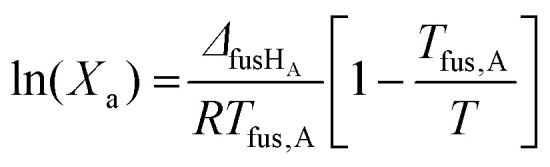


*X*_a_ = mole fraction of component A, *Δ*_fusH_A__ = heat required to melt one mole of A, *T*_fus,A_ = melting temperature of A.

Differential scanning calorimetry (DSC) is typically employed to obtain these melting temperature and enthalpy values. If the eutectic point of the experimental curve is at a lower temperature than that of the theoretical curve, the resulting mixture is referred to as a deep eutectic solvent (DES).^1^

The term ‘deep eutectic solvent’ is often applied loosely due to the absence of a universally accepted definition. To address this, Martins *et al.*^23^ proposed a more rigorous description, defining a DES as ‘a mixture of two or more pure compounds where the eutectic point temperature is significantly lower than that of an ideal liquid mixture, exhibiting notable negative deviations from ideal (Δ*T* > 0)’ (Fig. 1). Furthermore, the temperature reduction must be sufficient for the mixture to remain liquid at operating conditions over a range of compositions. If a mixture does not meet these criteria, the term ‘eutectic solvent’ is more appropriate.

DES can serve as solvents or excipients; however, they can also function as active pharmaceutical ingredients, in which case they are referred to as therapeutic deep eutectic solvents (TheDES).^1^ Some TheDES formulations have gained approval from regulatory bodies such as the Food and Drug Administration (FDA) and European Medicines Agency (EMA), with several products already available on the market. Notable examples include local anaesthetic creams, such as EMLA®, which is composed of lidocaine and prilocaine (1 : 1). In EMLA®, absorption of the anaesthetics is enhanced over that of the individual components.

When a DES consists of natural compounds, it is termed a natural deep eutectic solvent (NaDES). Choi *et al.*^24^ were amongst the first ones to realise the role of NaDES in plant metabolomics. NADES are abundant in living organisms and are assumed to play biological roles in organisms, alternative to water. For instance, honey is considered a NaDES, comprising glucose and fructose, which, while individually solid at room temperature, form a viscous liquid when combined.^25^Table 1 presents exemplary melting temperatures of various components, both in their pure forms and as part of eutectic mixtures.

**Table 1:** Melting temperatures of NaDES compared with those of their respective components^26,27^

NaDES compound 1/compound 2 (ratio)	NaDES melting temperature (°C)	Melting temperature of compound 1 (°C)	Melting temperature of compound 2 (°C)
Choline chloride/urea (1 : 2)	12	302	133
Menthol/octanoic acid (1 : 1)	−20	43	16

The performance of (Na)DES is frequently compared to that of ionic liquids (ILs). Discovered prior to DES, ILs are organic salts that remain liquid at temperatures below 100 °C.^28^ Their potential as green solvents has garnered significant attention due to their desirable properties, including non-flammability and non-volatility. However, despite these benefits, ILs present certain drawbacks, such as high toxicity, high production costs, complex synthesis, and poor biodegradability.^28^ In contrast, DES, and particularly NaDES, offer similar benefits to ILs while mitigating many of their disadvantages. The components of NaDES are often structurally simple and inexpensive, requiring minimal energy for synthesis or extraction.^18^ Furthermore, NaDES tend to be less toxic and environmentally less problematic, as they decompose readily without producing harmful by-products. This combination of low toxicity, biodegradability, and cost-effectiveness makes NaDES an appealing alternative to ILs in various applications.

### Composition of NaDES

NaDES are typically composed of at least two components, often involving a Lewis or Brønsted acid and base.^29^ Most NaDES described to date consist of ‘primary’ or biosynthetically primordial metabolites (PRIM), which can be grouped into five main categories based on their chemical nature: quaternary ammonium compounds, organic acids, amino acids, sugars and fatty acids.^30^

**Fig. 2:**
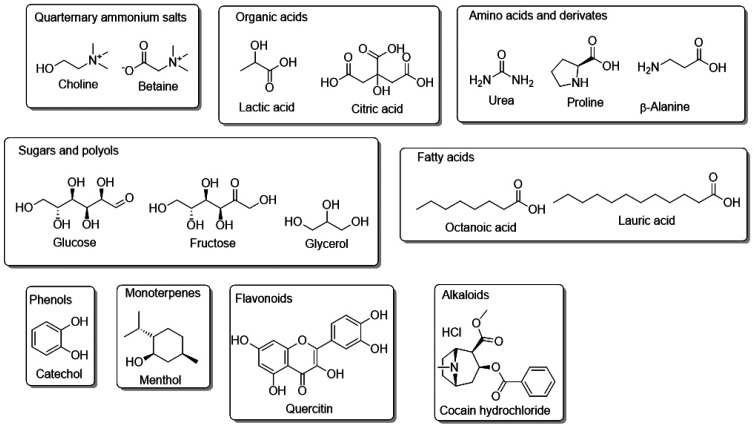
Structures of several NaDES components.

In addition to these primary metabolites, recent studies have identified more complex ‘specialised’ or biosynthetically advanced metabolites (HEVO) as potential NaDES components.^30–35^ Examples of these include flavonoids, monoterpenes, phenols and alkaloids, which offer expanded functionality and potential applications (Table 2 and Fig. 2). The versatility of NaDES is further highlighted by the vast number of metabolite combinations described in the literature, with most of these combinations detailed in Table 3.

**Table 2:** NaDES components^1,27,30–41^

Biosynthetically primordial metabolites (PRIM)					Biosynthetically more highly evolutionary metabolites (HEVO)			
Quarternary amines	Organic acids	Amino acids and derivates	Sugars and others polyols	Fatty acids	Phenols	Monoterpenes	Flavonoids	Alkaloids
Acetylcholine chloride	Acetic acid	*n*-Acetylcysteine	Adonitol(ribitol)	Capric acid	Caffeic acid	Camphor	Catechin	Cocaine hydrochloride
Betaine	Aconitic acid	β-Alanine	Butanols and butanediols	Lauric acid	Catechol	1,8-Cineole	Quercetin	Nicotine
Cholinebitartrate	Adipic acid	Arginine	*meso*-Erythritol	Myristic acid	Chlorogenic acid	Geraniol	Naringenin	
Choline chloride (ChCl)	Ascorbic acid	Cysteine	Fructose	Nonanoic acid	*o*-Cresol	Linalool	Naringin	
	Aspartic acid	Glycine	Galactose	Octanoic acid	Gallic acid	Menthol	Rutin	
	Citric acid	Histidine	Glucose	Oleic acid	Phenol	Thymol		
	Glutaric acid	Lysine	Glycerol	Stearic acid	Resorcinol			
	Glycolic acid	Methionine	Glycol		Resveratrol			
	Itaconic acid	Proline	Inositol		Rosmarinic acid			
	Lactic acid	Serine	Lactose		2,3-Xylenol			
	Levulinic acid	Tryptophane	Maltose					
	Maleic acid	Urea	Mannose					
	Malic acid	Valine	Propanols and propanediols					
	Malonic acid		Raffinose					
	Nicotinic acid		Rhamnose					
	Oxalic acid		Sorbitol					
	Phenylacetic acid		Sorbose					
	Propanoic acid		Sucrose					
	Sodium phytic acid		Trehalose					
	Succinic acid		Xylitol					
	Tartaric acid		Xylose					

**Table 3:** Selection of NaDES combinations

Combination type	Example	Ref.
Quaternary amine–organic acid	ChCl–lactic acid (1 : 1)	25
Quaternary amine–sugar	Betaine–glucose (5 : 2)	25
Organic acid–sugar	Malic acid–xylose (1 : 1)	25
Sugar–sugar	Fructose–sucrose (1 : 1)	25
Sugar–sugar–sugar	Glucose–fructose–sucrose (1 : 1 : 1)	25
Organic acid–amino acid	Citric acid–proline (1 : 3)	25
Amino acid–sugar	Proline–glucose (5 : 3)	25
Quaternary amine–organic acid–amino acid	Betaine–malic acid–proline (1 : 1 : 1)	25
Quaternary amine–organic acid–sugar	Betaine–oxalic acid–glucose (1 : 1 : 1)	25
Quaternary amine–sugar–amino acid	Betaine–sucrose–proline (1 : 1 : 1)	25
Quaternary amine–polyol–sugar	Betaine–inositol–raffinose (9 : 1 : 1)	25
Fatty acid–fatty acid	Dodecanoic acid–nonanoic acid (1 : 3)	38
Fatty acid–fatty acid–fatty acid	Octanoic acid–decanoic acid–dodecanoic acid (3 : 1 : 1)	38
Fatty acid–monoterpene	Oleic acid–menthol (1 : 2)	1
Monoterpene–monoterpene	Menthol–thymol (1 : 4)	1
Monoterpene–organic acid	Menthol–lactic acid (8 : 1)	42
Phenol–alkaloid–monoterpene	Phenol–cocaine–menthol	34
Quaternary amine–phenol	ChCl–resorcinol	31
Quaternary amine–flavonoid	ChCl–quercetin (6 : 1)	33

### NaDES characteristics

Most NaDES exhibit melting points below 100 °C, with many remaining liquid at room temperature. Generally, (Na)DES with melting points below 50 °C are considered particularly advantageous for practical use.^28^Table 4 highlights the characteristics of various NaDES, including their melting points, demonstrating the suitability of these solvents for diverse applications.

**Table 4:** Melting temperature, viscosity and polarity parameters of various NaDES

NaDES	*T*_M_ [°C]	Polarity [kcal mol^−1^]	Viscosity [mPa s]	Ref.
ChCl/glycerol (1 : 2)	−40	58	259 (25 °C)	28 and 39
ChCl/malonic acid (1 : 1)	10	45	1084 (25 °C)	28, 37 and 43
ChCl/urea (1 : 2)	12	—	750 (25 °C)	28 and 43
ChCl/glucose (1 : 1)	—	50	34 400 (50 °C)	28 and 39
Octanoic acid/lauric acid (3 : 1)	9	—	7 (25 °C)	38
Glucose/citric acid (1 : 1)	—	48	5500 (25 °C)	39 and 44
Menthol/octanoic acid (1 : 1)	−20	61	12 (25 °C)	27 and 45

Polarity is a key characteristic of a NaDES as a solvent. NaDES with lipophilic properties, such as those based on fatty acids and monoterpenes, are particularly effective at solubilising lipophilic or low-polarity compounds.^1,38^ In contrast, hydrophilic NaDES exhibit a wide range of polarities, making them suitable for dissolving both hydrophilic and lipophilic substances.^30^ The polarity of NaDES is frequently assessed using spectroscopic methods, particularly through solvatochromic probes like Nile red (NR). This method involves exposing the solvatochromic probe to the solvent of interest and measuring UV-spectrum. From *λ*_max_, the molar transition energy (*E*_NR_) can be calculated using *E*_NR_ = 28 591·*λ*_max_^−1^.^39^

Acid-based NaDES are the most polar, with *E*_NR_ values ranging from 44.8 to 48.3 kcal mol^−1^, placing them in the same polarity range as water (*E*_NR_ = 48.2 kcal mol^−1^) or even higher. Sugar-based NaDES exhibit lower polarities (*E*_NR_ = 49.7 to 50.7 kcal mol^−1^), comparable to that of methanol (*E*_NR_ = 51.9 kcal mol^−1^). Alcohol-based NaDES are the least polar, with *E*_NR_ values ranging from 49.6 to 58.6 kcal mol^−1^.^39^ The polarity of a NaDES can be modulated by adjusting the molar ratios of its components. For instance, in a ChCl/citric acid mixture, the *E*_NR_ decreases as the ratio shifts from 2 : 1 to 1 : 1, indicating a change in polarity. Conversely, the *E*_NR_ of ChCl/glycerol increases when the molar ratio changes from 1 : 3 to 1 : 2. Additionally, the introduction of water into a NaDES lowers *E*_NR_ values, thereby enhancing the mixture's polarity.^39^

**Fig. 3:**
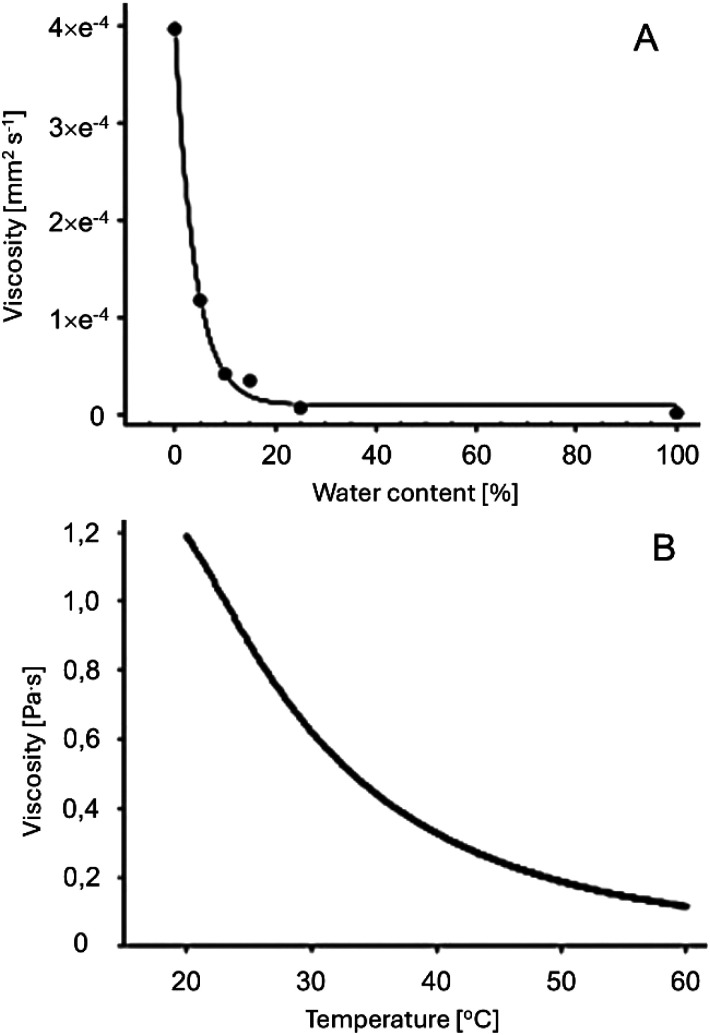
Viscosity of the eutectic mixture glucose/ChCl/water (2 : 5 : 5) as a function of water content (A) and temperature (B). This figure has been adapted from ref. 25 with permission from Elsevier, copyright 2013.

Viscosity, another critical factor in the application of NaDES, is often high, as highlighted in Table 4. Polar NaDES tend to exhibit higher viscosity than their nonpolar counterparts. Viscosity can be modulated by increasing temperature (Fig. 3), altering the component ratios, or introducing a third element, such as water or alcohols like glycerol or ethanol.^39^

### Chemical interactions within NaDES

NaDES typically comprise at least one hydrogen bond donor (HBD) and one hydrogen bond acceptor (HBA). Interactions between them, primarily hydrogen bonding, occasionally reinforced by van der Waals forces, lead to the melting point decrease.^27,39^

To elucidate the interactions within ChCl-based eutectic mixtures, various analytical techniques have been employed, including crystallography, mass spectrometry, infrared spectroscopy, and nuclear magnetic resonance (NMR), with a particular focus on diffusional NMR techniques like diffusion ordered spectroscopy (DOSY).^46^ Computational approaches, such as density functional theory (DFT), have also been utilised to provide deeper insights into the structural characteristics of these mixtures.^47^ These revealed that the electronegative chloride anion can form interactions with one or more HBD. These interactions result in a redistribution of the negative charge, weakening the ionic attraction between the choline cation and the chloride counterion. Consequently, the introduction of a HBD disrupts the HBA network, leading to a reduction in the overall melting point of the eutectic mixture.^46^

**Fig. 4:**
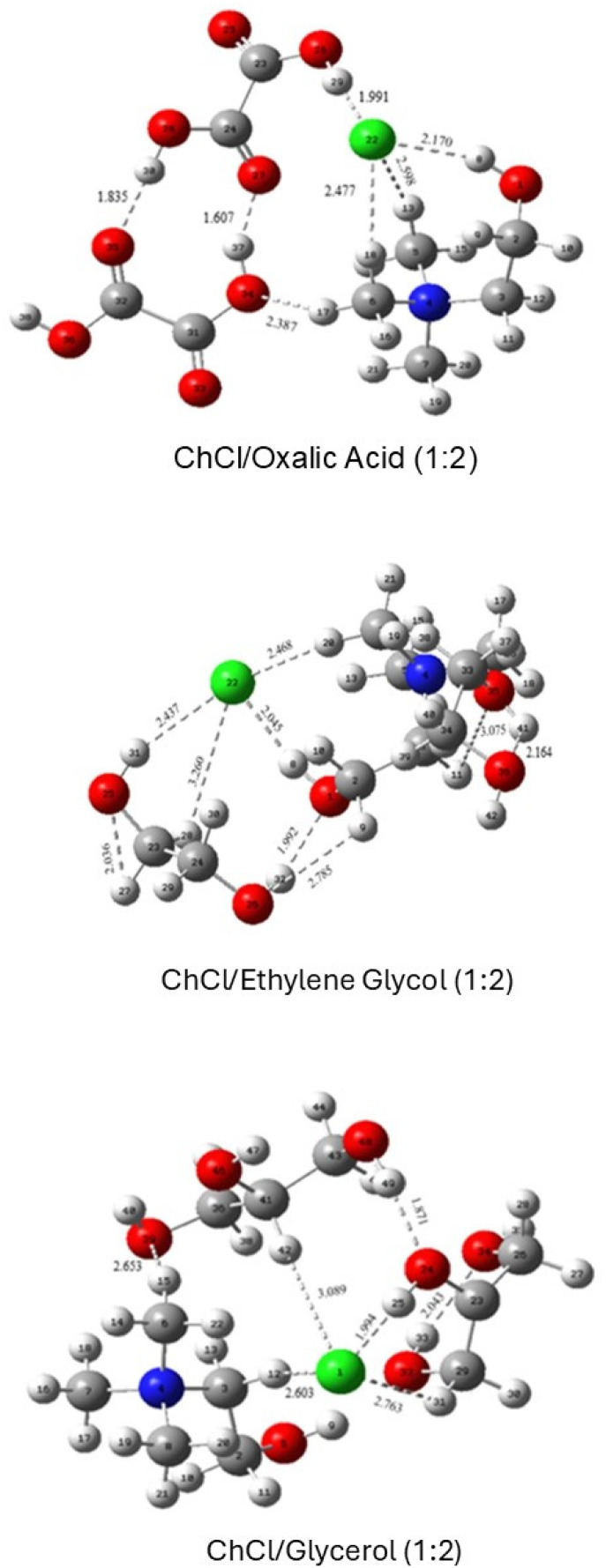
Supramolecular structures of some DES. This figure has been adapted from ref. 47 with permission from Elsevier, copyright 2021.

The hydrogen bond between the chloride counterion and the HBD is not the sole interaction that influences the melting point. As illustrated in Fig. 4, several additional interactions, including intermolecular hydrogen bonds between the hydroxyl group of the choline cation and the HBD, as well as interactions between different HBD play a role. Also, intramolecular hydrogen bonds are important.^47^

Collectively, these interactions create a complex network of bonds within the eutectic system influencing their melting points and viscosities.^46,48^

D'Agostino *et al.*^49^ investigated these interactions using diffusional NMR, which allowed them to determine the individual diffusion coefficients. In mixtures such as ChCl/glycerol (1 : 2), the choline cation diffuses more slowly than the associated HBD. Conversely, in ChCl/malic acid (1 : 2) mixtures, malic acid tends to dimerise and diffuse more slowly than the choline cation. These findings underscore that the nature of the HBD plays a significant role in determining NaDES properties.

The Hole Theory, initially proposed by Abbott *et al.*,^50^ posits that the physical properties of a material, such as viscosity and diffusion, can be explained by the presence of microscopic holes or voids within the material. These empty spaces that arise due to density fluctuations within the material are dynamic, continually forming and disappearing due to Brownian motion. Their size, shape and distribution are influenced by the temperature and the nature of the material. In a liquid or amorphous solid, regions of higher density have fewer or smaller holes, while regions of lower density have more or larger holes. As the number or size of holes increases, the material's viscosity may decrease and diffusion tends to be easier because particles have more space to move through the material. During the melting process, the formation of holes is more pronounced, as the material's structure becomes more disordered and the density decreases.

The investigation of chemical interactions within NaDES offers a pathway to understanding and predicting the optimal molar ratios between component molecules. For example, Sun *et al.*,^26^ elucidated the ideal 1 : 2 mole ratio between ChCl and urea through the application of molecular dynamics simulations. Their study examined the interactions between cations and urea, anions and urea, cations and anions, as well as urea–urea bonds across various molar ratios. They found that the 1 : 2 ratio not only maximised energetic efficiency but also achieved the most favourable charge distribution, highlighting the precision required in designing eutectic mixtures for specific applications.

Eutectic mixtures do not always involve an ionic species acting as a HBA, such as ChCl. Honey, for example, is a naturally occurring eutectic mixture primarily composed of fructose, glucose and water, all of which can function as both HBA and HBD. Brudzynski *et al.*^51^ investigated the molecular interactions within honey by combining techniques such as UV spectrometry, dynamic light scattering, scanning electron microscopy, and mathematical modelling. Their findings suggest that the high concentration of sugar in honey creates conditions similar to the congestion of macromolecules in the cell. Most of the water is bound to the sugars and is not available, causing the macromolecules in honey to congregate and form stable micron-sized particles. This extremely crowded environment encourages non-specific and non-covalent interactions between macromolecules, which collectively contribute to the unique physicochemical behaviour of honey.

### Preparation of NaDES

To date, seven distinct techniques have been identified for the preparation of NaDES, as summarised in Table 5.^1^ The selection of the most suitable method is largely determined by the specific properties of the eutectic components, with particular attention to their sensitivity to heat. This is a critical consideration, as exposure to elevated temperatures can lead to the degradation of certain compounds or trigger reactions with water, potentially resulting in the formation of undesirable by-products. Therefore, careful evaluation of the thermal stability of the components is essential when choosing the appropriate preparation technique.

**Table 5:** NaDES preparation methods^1^

Method	Description	Benefits	Limitations
**Starting from the pure compounds**			
Heating and stirring	Stirring and moderate heating	Simplicity	Possible thermal degradation
Grinding	Mixing using a mortar and pestle	Suitable for heat-sensitive materials	No temperature control during the process
Twin screw extrusion	Continuous mixing using a twin-screw extruder	Scalability	Specialised equipment needed
Microwave irradiation	As above, using microwave heating	Speed, simplicity	Possible thermal degradation
Ultrasound-assisted preparation	As above, using ultrasounds	Speed, simplicity	

**Starting from solutions of the compounds**			
Lyophilisation	Lyophilisation of frozen aqueous solutions of the NaDES components	Suitable for heat-sensitive materials	Not suitable for NaDES containing volatile compounds
Vacuum evaporation	Distillation of water from aqueous solutions of the NaDES components under reduced pressure	Suitable for heat-sensitive materials	Not suitable for NaDES containing volatile compounds

### NaDES stability

**Fig. 5:**
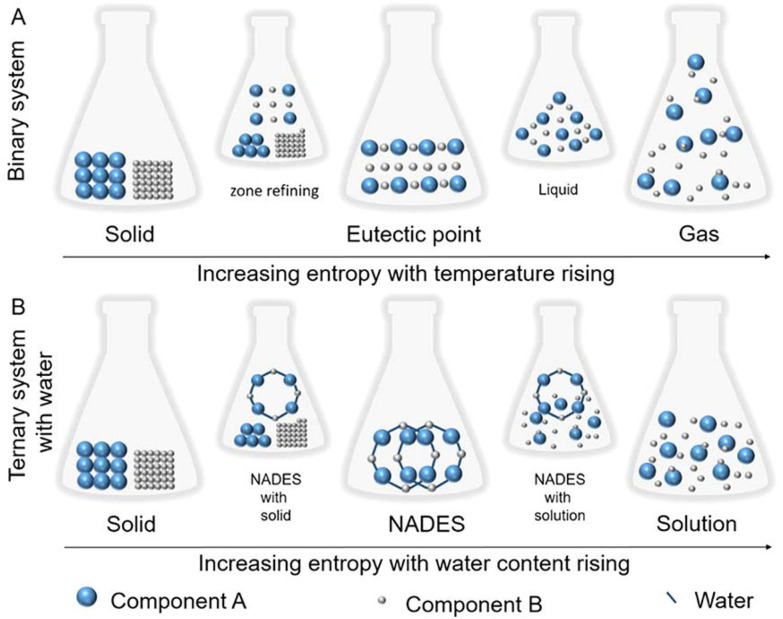
Kinetic energy models illustrating (A) the effect of increasing temperature on a binary NaDES and (B) the impact of water addition on a ternary NaDES. This figure has been adapted from ref. 30 with permission from American Chemical Society, copyright 2018.

Owing to the supramolecular structure formed by non-covalent interactions between its components, NaDES are generally highly stable entities. However, certain parameters, such as temperature and water content, can influence their supramolecular arrangement, thereby affecting their physicochemical properties. To explain this phenomenon, Liu *et al.*^30^ proposed kinetic energy models (Fig. 5).

The effect of water on the supramolecular structure of NaDES is typically investigated using proton nuclear magnetic sesonance (^1^H NMR) or nuclear Overhauser effect spectroscopy (NOESY). For instance, Dai *et al.*^52^ recorded the ^1^H NMR signal shifts of a 1,2-propanediol/ChCl/water mixture (1 : 1 : 1) at varying dilutions in deuterium oxide (D_2_O). Their findings indicate that dilutions of 25–50% D_2_O progressively disrupt hydrogen bonds. According to their analysis, the supramolecular structure of NaDES remains stable up to 50% water content, beyond which the components dissociate into their free forms. These observations align with subsequent studies by Gabriele *et al.*^53^ and Spaggiari *et al.*,^54^ which examined mixtures of ChCl with glycols and betaine with glycerol, respectively. However, Gabriele *et al.* observed molecular interactions persisting up to 75% water content. These conclusions further support the kinetic models proposed by Liu *et al.*^30^

NaDES exhibit stability in the liquid state over a broad temperature range, providing a wide operational window. Savi *et al.*^55^ reported that ChCl/lactic acid (1 : 1) and lactic acid/glucose (5 : 1) remained stable between −68 °C and 72 °C, and −68 °C and 25 °C, respectively. Notably, the presence of water enhances stability at lower temperatures, allowing lactic acid/glucose/water (5 : 1 : 3) to remain stable down to −75 °C. These findings are consistent with those of Santana *et al.*,^56^ who investigated xylitol/malic acid/water (1 : 1 : 10), xylitol/citric acid/water (1 : 1 : 10), and malic acid/citric acid/water (1 : 1 : 10). These mixtures exhibited water loss at approximately 100 °C and decomposed at 160 °C, 165 °C, and 180 °C, respectively. However, the decomposition temperature of NaDES can vary depending on the presence of other components in solution. For instance, while urea-containing NaDES typically decompose above 200 °C, Hu *et al.*^57^ observed partial decomposition of urea *via* reaction with tri-carbonyl compounds, enabling urea to participate in the synthesis for which it serves as a solvent (Fig. 16).

The long-term stability of NaDES has only recently been characterised. Spaggiari *et al.*^54^ reported that the physicochemical properties of most NaDES remained stable over a period of at least twelve months. However, certain NaDES, such as betaine/lactic acid (1 : 1), exhibited significant changes in viscosity, conductivity, and polarity over this timeframe, likely due to moisture absorption. To ensure optimal preservation, NaDES should be stored in airtight containers.

As solvents, NaDES are expected to be reused multiple times, necessitating stability over several cycles. Singh *et al.*^58^ demonstrated the stability of ChCl/urea (1 : 2) across five reaction cycles, with consistently high yields. This aspect will be explored further in the subsequent section.

### NaDES recovery and reuse

Solvent recycling is a crucial aspect of reducing the environmental impact associated with industrial processes.^59^ For most organic solvents, destillative recycling is straight-forward, provided the compounds in solution are non-volatile. The non-volatile nature of most NaDES makes their recycling more complex. To address this, several alternative techniques have been developed, which often simplify the purification steps compared to conventional methods.^60^

**Fig. 6:**
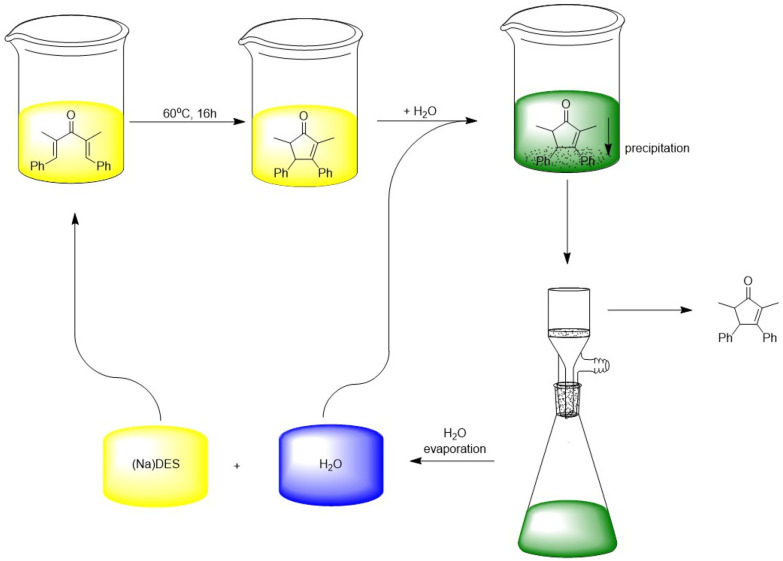
Recycling process for (Na)DES and water involved in the thermal cyclisation of divinyl ketone. This figure has been adapted from ref. 61 with permission from Royal Society of Chemistry, copyright 2020.

Many recycling methods for NaDES involve the separation of the reaction products by precipitation following the addition of an anti-solvent, typically water.^60^ Since most synthesised products are soluble in organic solvents, while NaDES are usually water-soluble, the products precipitate upon water addition, allowing for their separation from NaDES through filtration. Nejrotti *et al.*^61^ have extensively detailed this process, particularly in the context of cyclising divinyl ketones in NaDES (Fig. 6). Their method enables the recovery and reuse of both NaDES and water across four reaction cycles, all while maintaining an acceptable overall yield, ranging from 97% in the first cycle to 65% in the last one. This approach results in pure compounds, eliminating the need for column chromatography purification, thus streamlining the overall process.

Singh *et al.*^58^ employed a similar approach to optimise the synthesis of imines and bisamides from primary amines *via* the Ugi reaction in ChCl/urea (1 : 2), catalysed by 2-iodoxybenzoic acid (IBX) as the oxidant. The product and catalyst were efficiently recovered through a simple procedure involving the addition of water, followed by filtration. The resulting precipitate, containing the organic phase with both product and catalyst, was then treated with ethyl acetate. In this step, the product dissolved in the ethyl acetate, while the catalyst precipitated out, allowing for its recovery and subsequent oxidation for reuse. This method was successfully repeated over five cycles, demonstrating catalytic stability between 80% and 70% across the first four cycles before declining below 60% in the final cycle. The NaDES remained stable throughout all five cycles.

When precipitation-based separation is not feasible, products can be extracted using a liquid–liquid extraction method with organic solvents. Although this approach involves the use of organic solvents, it often simplifies the purification process compared to conventional chromatographic methods. For instance, Di Gioia *et al.*^62^ demonstrated that extracting their product with ethyl acetate enabled the NaDES to be reused up to four times without affecting the reaction yield, which remained above 80%.

The complexity of separation processes can increase when NaDES is used as an extraction solvent, especially since the extracted metabolites are often water-soluble and do not precipitate upon the addition of water. To recover the phenolic metabolites from *Carthamus tinctorius* L., Dai *et al.*^63^ used a chromatographic resin (Diaion HP-20) that retained the phenolic derivatives while eluting the NaDES with water.

Alternative methods described in the literature include crystallisation, membrane filtration, solid–liquid extraction, liquid–liquid extraction, short-path distillation, supercritical fluid extraction, density difference separation, and centrifugal partition chromatography.^60,64^ Some of these techniques have achieved NaDES recoveries exceeding 90%. However, many require substantial quantities of additional aqueous and ethanolic solvents, leading to high energy consumption and increased costs. Consequently, selecting an appropriate recovery method involves careful consideration of the specific properties of the NaDES, the nature of the conversion or extraction process, the characteristics of the target compound, as well as the associated energy requirements and equipment costs.^60^

Volatile NaDES, primarily composed of monoterpenes, have also been shown to be highly effective for extraction and can be recovered and reused following evaporation. Strzemski *et al.*^65^ utilised menthol/thymol (5 : 5) and thymol/camphor (6 : 4) mixtures to efficiently extract isoquinoline alkaloids from *Chelidonium majus*. The alkaloid yields were significantly higher, up to twice as high for some metabolites, when using NaDES compared to conventional extraction methods involving acidified water or methanol, followed by dichloromethane (DCM) or chloroform.

**Fig. 7:**
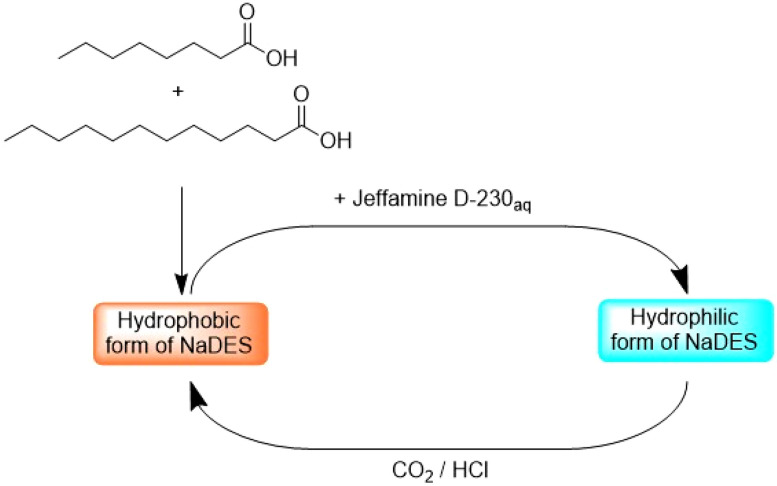
Reversible polarity switch in NaDES. This figure has been adapted from ref. 66 with permission from Royal Society of Chemistry, copyright 2018.

Sed *et al.*^66^ have introduced an innovative method that leverages the hydrophilicity-switching property of a fatty acid-based NaDES. The polarity of this unique NaDES can be altered *in situ* to hydrophilic by adding water and polyamines such as Jeffamine D-230, while the introduction of CO_2_ or a strong acid such as HCl reverts it back to its hydrophobic form (Fig. 7). This reversible transformation allows for the selective extraction of compounds based on their nature. After extraction, the NaDES is modified *in situ* to induce the precipitation of the extracted metabolites, facilitating their recovery and further processing. The method described is a real tool that can be used in different ways, either in ‘forward mode’ with initial contact with the hydrophobic phase and recovery of the hydrophilic solutes and sequential recovery of the hydrophobic solutes after switching, or in the reverse process in ‘reverse mode’. The tool was applied to microalgal biomass to evaluate the extraction efficiency of proteins, carbohydrates and neutral lipids. A mode can be selected according to the interest in certain compounds, and the process can be improved by additional steps, such as bead beating or microwaving to achieve the desired selectivity.

This technique exemplifies the evolving strategies in solvent recovery and reusability, highlighting the potential of NaDES to adapt to various industrial applications.

## Pharmaceutical applications of NaDES

**Fig. 8:**
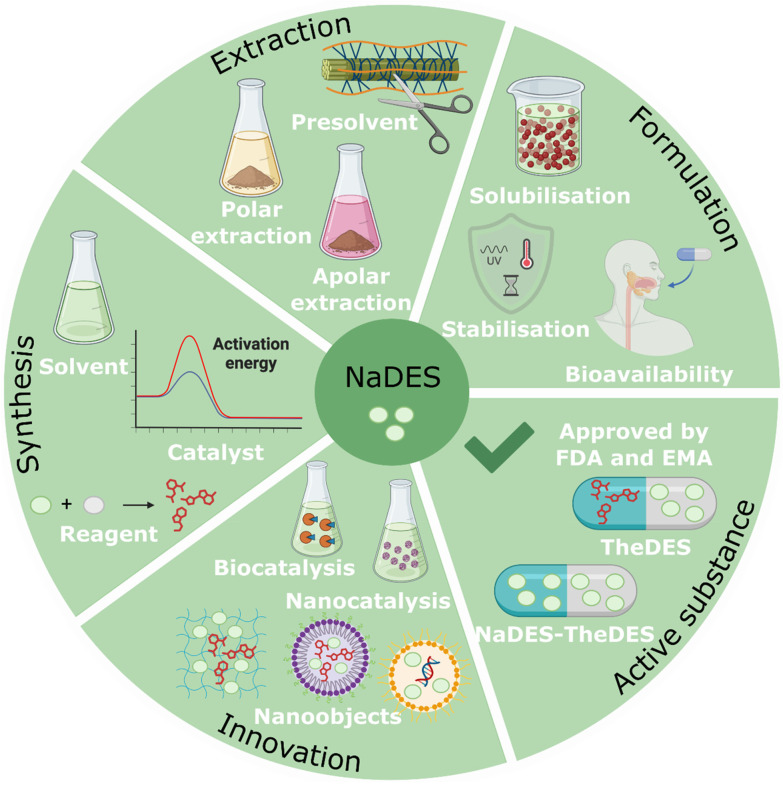
Applications of NaDES in the pharmaceutical industry.

Research into NaDES is progressing rapidly, uncovering a broad spectrum of applications in the synthesis and extraction of active ingredients, as well as in their formulation.^19^ Additionally, NaDES can function as active ingredients themselves, referred to as TheDES, and are being investigated for potential applications in emerging fields such as biotechnology and nanotechnology (Fig. 8).

### NaDES in synthesis

**Fig. 9:**
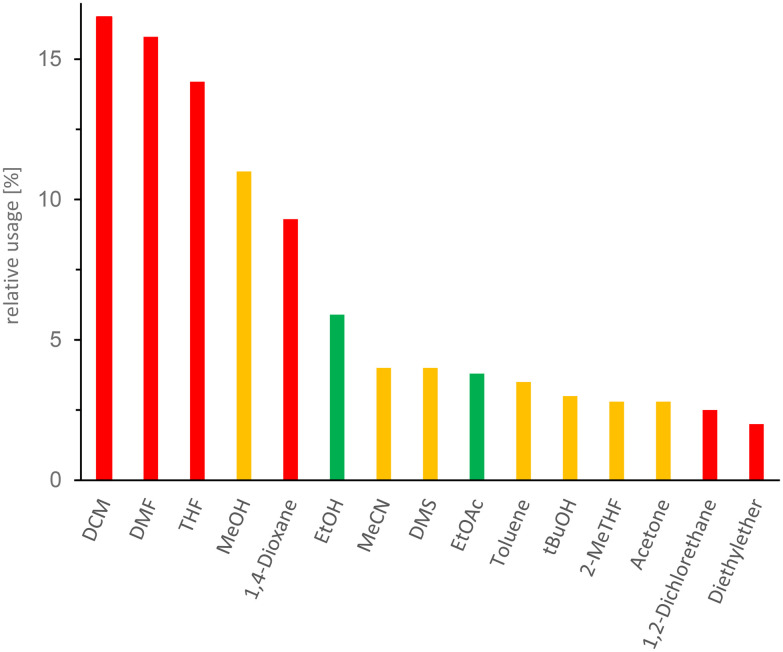
Histogram of the most abundant solvents used in the pharmaceutical industry. The colour coding follows the EHS-evaluation of organic solvents: green = few issues, orange = problematic, red = dangerous. DCM (dichloromethane), DMF (*N*,*N*-dimethylformamide), THF (tetrahydrofuran), MeOH (methanol), EtOH (ethanol), MeCN (acetonitrile), DMS (dimethyl sulfide), EtOAc (ethyl acetate), tBuOH (*tert*-butanol), 2-MeTHF (2-methyltetrahydrofuran). This figure has been adapted from ref. 67 with permission from American Chemical Society, copyright 2021.

Despite the growing interest in greener alternatives, conventional organic solvents continue to dominate chemical synthesis, particularly in medicinal chemistry. A study conducted by Jordan *et al.*^67^ provided a comprehensive overview of solvent use, serving as a baseline for solvent usage in typical medicinal chemistry research. Fig. 9 highlights some common solvents.

While these solvents are widely used for their effectiveness in various chemical processes, they pose significant risks to human health.^68^ DCM, 1,4-dioxane, *N*,*N*-dimethylformamide (DMF) and tetrahydrofuran (THF) are particularly concerning, as they are classified under the category of carcinogenic–mutagenic–reprotoxic (CMR) substances. Specifically, DCM, 1,4-dioxane and THF are suspected human carcinogens, falling under category 2 carcinogens. DMF is even more troubling, as it is classified as a category 1B reprotoxic (presumed risk of reproductive toxicity in humans).^68^

THF and methanol are primarily known for their neurotoxic effects. THF is recognised for its narcotic properties, which can impair neurological function, while methanol is notorious for its toxicity to the optic nerve, potentially leading to blindness or other severe visual impairments.

NaDES have emerged as promising candidates to replace these traditional solvents. One of the key advantages of NaDES lies in their ability to form hydrogen bonds with solutes, which not only facilitates dissolution but also stabilises transition states during chemical synthesis. This unique property enhances the efficiency of synthetic processes, offering a safer and more sustainable alternative to hazardous organic solvents.

NaDES also provide significant benefits during purification processes. When water is added to a polar NaDES, it often induces the precipitation of organic products.^60^ This phenomenon simplifies purification operations by eliminating the need for additional solvents, thereby reducing the overall solvent load and minimising environmental impact. This ability to streamline purification not only makes NaDES an attractive alternative from a green chemistry perspective but also improves the practicality and cost-effectiveness of chemical processes.

**Fig. 10:**
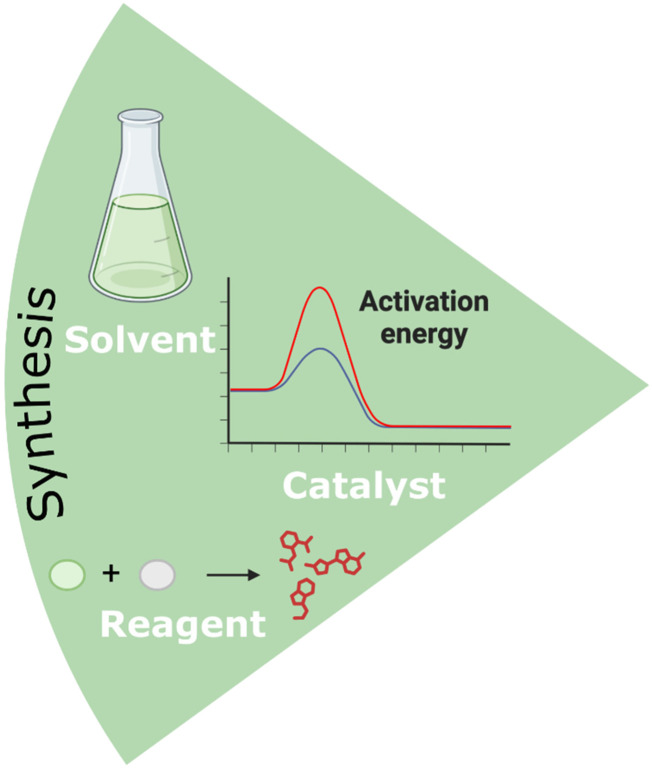
Application of NaDES in the synthesis of pharmaceutical compounds.

NaDES can also play a dual role as solvents and catalysts, or as solvents and reagents. ChCl-based NaDES have been the most extensively studied for these applications (Fig. 10).^46^

#### NaDES as reaction medium

**Fig. 11:**
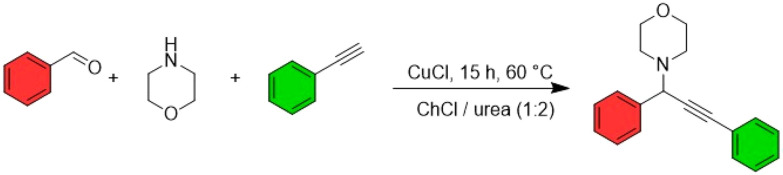
Synthesis of propargylamines. This figure has been adapted from ref. 46 with permission from Multidisciplinary Digital Publishing Institute, copyright 2021.

The application of NaDES in the synthesis of active pharmaceutical ingredients (APIs) is gaining traction as researchers explore their potential to replace conventional solvents. A notable example of this is the work by Abtahi and Tavakol,^69^ who recently developed a novel method for synthesising propargylamines using ChCl/urea (1 : 2) as the solvent (Fig. 11). In their process, morpholine, phenylacetylene, and aromatic aldehydes were reacted in the presence of a copper(i) chloride (CuCl, 5 mol%) catalyst. Propargylamines serve as essential building blocks in drug design, with applications in the synthesis of various pharmaceuticals, including rasagiline, a drug commonly used in the treatment of Parkinson's disease. Traditionally, the synthesis of propargylamines for rasagiline production has relied on acetonitrile, a solvent that is acutely toxic if swallowed, inhaled, or comes into contact with the skin.^70^ The PMI for the synthesis step described in the rasagiline production patent is 6.0. The alternative method proposed by Abtahi and Tavakol yields a comparable PMI (7.8), with reactants shown in Fig. 11. Notably, the solvent used in their approach is less hazardous, and the reaction proceeds *via* a one-pot process, simplifying synthesis. Adapting this method for rasagiline production and evaluating its scalability could therefore be a valuable avenue for further research and industrial application.

**Fig. 12:**
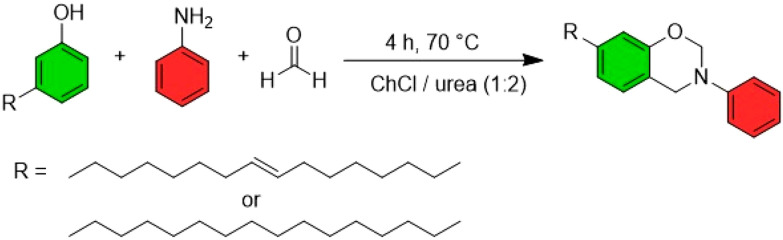
Benzoxazine synthesis. This figure has been adapted from ref. 46 with permission from Multidisciplinary Digital Publishing Institute, copyright 2021.

ChCl/urea (1 : 2) has also been used as a solvent for the synthesis of benzoxazines, promising candidates as antilipidemic agents. Behalo *et al.*^71^ explored the preparation of benzoxazines from cardanol, a phenolic lipid extracted from cashew nuts or *Ginkgo biloba*, which attracts considerable interest for its possible applications in pharmaceutical chemistry. They developed a Mannich-type condensation reaction involving cardanol, aniline and formaldehyde in ChCl/urea (1 : 2), achieving very satisfactory yields (81–88%) (Fig. 12).

**Fig. 13:**
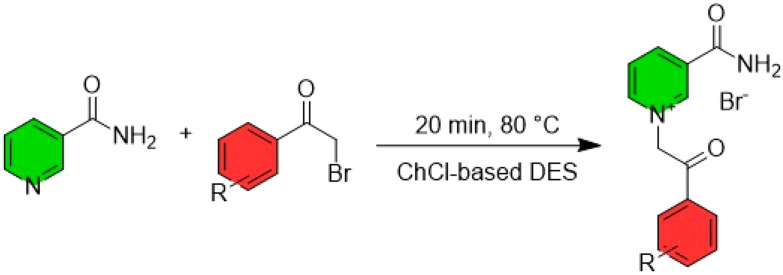
Synthesis of quaternary nicotinamide derivatives. This figure has been adapted from ref. 46 with permission from Multidisciplinary Digital Publishing Institute, copyright 2021.

Furthermore, NaDES have proven to be effective in the synthesis of derivatives of nicotinamide (vitamin B3). These quaternary derivatives are of significant interest due to their antimicrobial, fungicidal, and cytotoxic properties. In a study by Bušić *et al.*^72^ the nucleophilic substitution of 2-bromoacetophenone by nicotinamide was carried out under microwave conditions using a ChCl-based NaDES (Fig. 13). Whether ChCl was combined with urea (1 : 2) or organic acids such as malic acid (1 : 1) or lactic acid (1 : 2), the resulting quaternary derivative was obtained in good yield, demonstrating the versatility and efficiency of NaDES in this synthetic process.

#### NaDES as catalyst

**Fig. 14:**
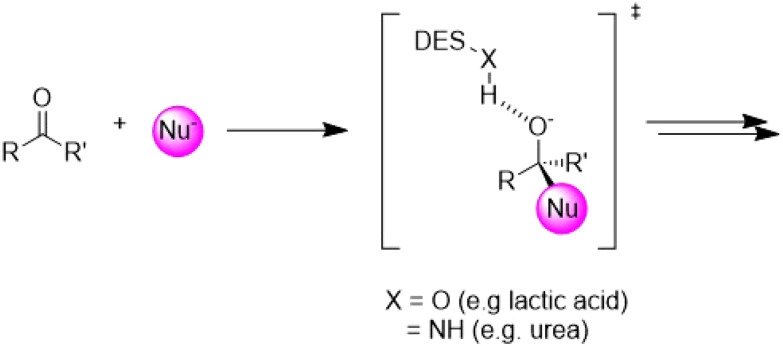
Stabilisation of the oxyanion intermediate *via* H-bonding to the DES. This figure has been adapted from ref. 46 with permission from Multidisciplinary Digital Publishing Institute, copyright 2021.

Remarkably, some NaDES can also simultaneously serve as the reaction medium and as catalytically active agent. Various catalytic mechanisms have been proposed, primarily revolving around the formation of hydrogen bonds between the reactants and the NaDES (Fig. 14). To date, reactions such as Michael additions, transesterifications, and condensations have been successfully developed, all of which involve an electrophilic activation of a carbonyl group hydrogen bonding with the NaDES.^46^

Zamani and Khosropour^73^ performed a condensation followed by a Michael addition, catalysed by a ChCl/urea (1 : 2), to synthesise novobiocin derivatives. Novobiocin, an aminocoumarin secondary metabolite produced by various *Streptomyces* species, was originally marketed as albamycin in the 1960s as an antibiotic but was later withdrawn due to its insufficient activity. However, its aminocoumarin and dihydrocoumarin derivatives are currently under investigation for their cytotoxic, antibiotic, and anti-inflammatory properties. Traditional synthetic methods for these derivatives rely on organic solvents, particularly DMF.^74^ In contrast, Zamani and Khosropour developed the first multi-component, continuous-flow, cascade reaction to produce 3-aminohexahydrocoumarins using ChCl/urea as both solvent and catalyst. In addition to producing a higher yield (93%) than the reaction with DMF (67%), this novel approach demonstrated a better RME (64%) compared to the previous method (69%). However, the reactant concentrations in the NaDES are relatively low, necessitating a larger volume of solvent than in the conventional method, which accounts for the latter's advantage in terms of PMI. One potential solution could be the recycling of the NaDES. The traditional DMF process employed more functionalised reactants, resulting in a higher AE. The continuous-flow, multi-component method utilised readily available reagents and was easily scalable, affording 3.8 mg (91%) of product.

**Fig. 15:**
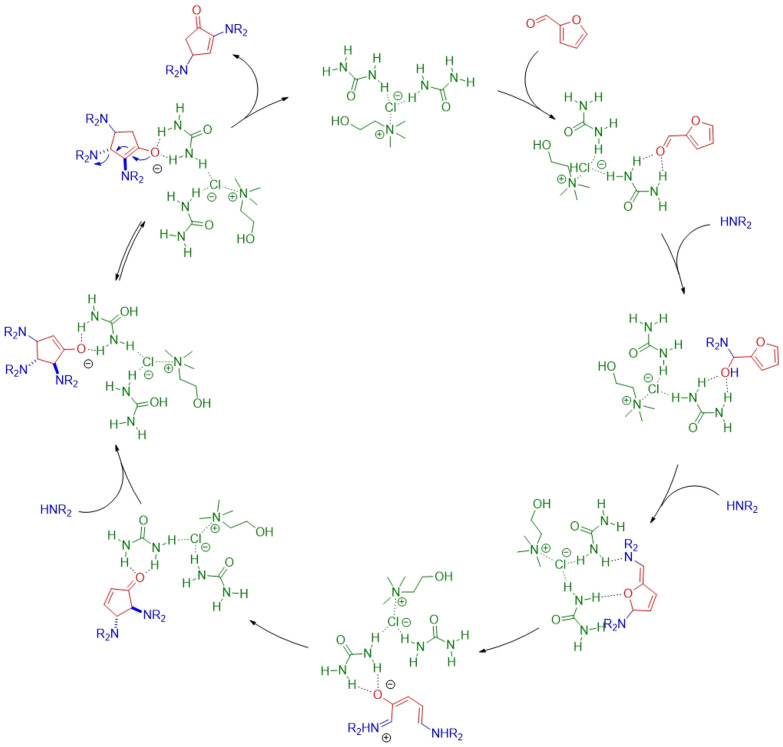
Proposed mechanism for the cyclopentenone functionalisation reaction. This figure has been adapted from ref. 62 with permission from Multidisciplinary Digital Publishing Institute, copyright 2018.

Additionally, the catalytic role of NaDES in the functionalisation of cyclopentenones has been elucidated by Di Gioia *et al.*^62^ In their proposed mechanism, the urea component of the NaDES activates the carbonyl group, initiating the reaction (Fig. 15). The hydrogen-bond-donating capacity of urea plays a crucial role throughout the process by stabilising the reaction intermediates. Finally, urea facilitates the formation of an enolic intermediate in the last step. The method developed by Di Gioia *et al.* not only employs a non-toxic, green solvent and catalyst but also eliminates the need for post-reaction purification. In addition, they recycled the solvent over five cycles without a significant reduction in yield. Since the solvent contributes substantially to the PMI, its recycling reduces the PMI from 6.1 to 2.3 (calculated over five cycles, accounting for yield reduction). This advancement is particularly noteworthy, as existing synthetic strategies for functionalising cyclopentenones typically rely on expensive catalysts and toxic solvents. For example, one approach employs a rare-earth element catalyst in an organic solvent, achieving a yield of >99%.^75^ As RME is highly dependent on yield, this reaction exhibits a high RME (93%), surpassing that of the NaDES method (89%). The organometallic method also demonstrated a favourable PMI (2.9). While green metrics serve as valuable indicators, this example also highlights their limitations regarding the nature of the compounds, catalysts, and reaction conditions involved. Notably, the reaction developed by Di Gioia utilised a readily accessible organic catalyst, proceeded six times faster, and remained easily scalable while maintaining a good yield (87%).

#### NaDES as reactant

**Fig. 16:**
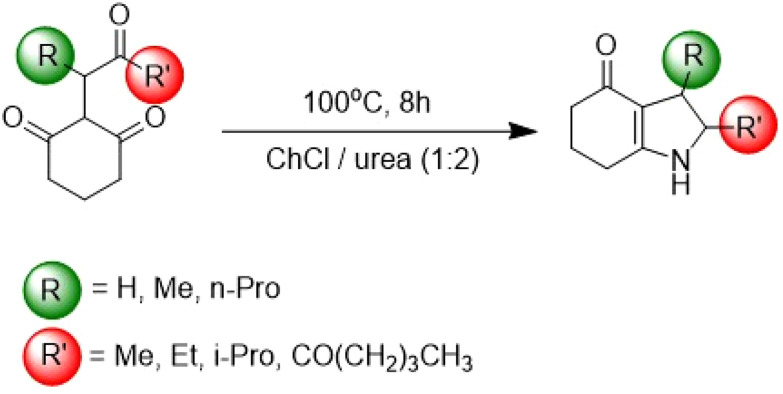
Synthesis of pyrrole derivatives. This figure has been adapted from ref. 46 with permission from Multidisciplinary Digital Publishing Institute, copyright 2021.

The constituent elements of NaDES can function as reactants in a chemical reaction, where they may be partially incorporated into the reaction product. An illustrative example is the use of ChCl/urea (1 : 2) as a nitrogen source in the synthesis of pyrrole derivatives, which are promising antimicrobial drug candidates.^62^ In the study by Hu *et al.*^57^ a condensation reaction between a tricarbonyl compound and ammonia—generated *in situ* from the ChCl/urea solvent—was observed (Fig. 16). This reaction proceeds efficiently at 100 °C, which facilitates the partial thermal decomposition of urea. Almost all resulting pyrrole derivatives were obtained in good to high yields depending on the starting triketone (71–93%).

**Fig. 17:**
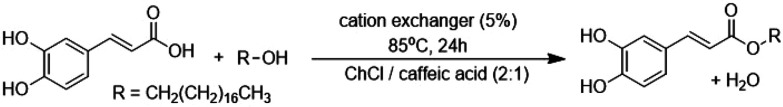
Caffeoyl ester synthesis. This figure has been adapted from ref. 46 from Multidisciplinary Digital Publishing Institute, copyright 2021.

One of the components of NaDES can also be fully incorporated into the final product during a reaction. Wang *et al.*^76^ described such a process in the production of caffeoyl esters from fatty alcohols using a NaDES composed of ChCl and caffeic acid in a 2 : 1 ratio, with a cation exchange resin serving as the catalyst (Fig. 17), achieving a high yield (91%). These esters are of significant interest because, by increasing the bioavailability of caffeic acid, they enhance its antioxidant, anti-inflammatory, antimicrobial, and anti-neoplastic properties.

**Fig. 18:**
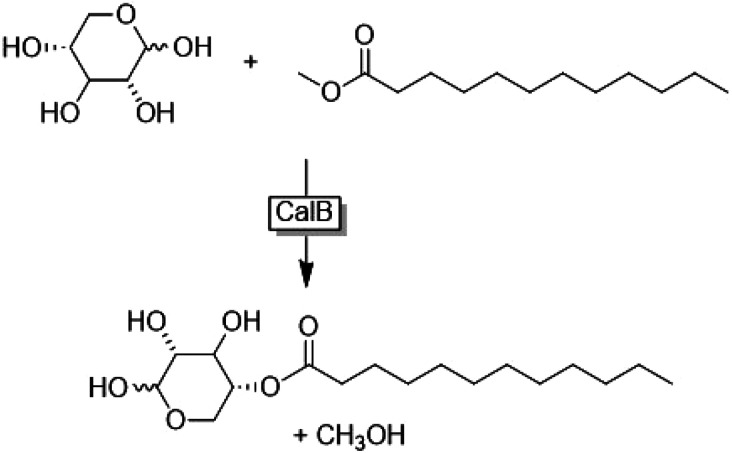
Enzymatic (CalB = lipase B from *Candida antarctica*) synthesis of fatty acid-carbohydrate esters in homogeneous phase. This figure has been adapted from ref. 77 with permission from Elsevier, copyright 2016.

In another example Siebenhaller *et al.*^77^ demonstrated the enzymatic synthesis of glycolipids using ChCl/carbohydrate-based DES (Fig. 18). These DES were sufficiently hydrophobic to solubilise fatty acid vinyl esters, thereby enabling the synthesis in a homogeneous phase. To prevent viscosity from lowering the yield of the reaction, mixing should be kept constant. This finding provides access to a novel class of glycolipids, offering new opportunities to develop their use as targeting agents in drug delivery or immunostimulants.^78,79^

Overall, NaDES exhibit significant potential in the synthesis of pharmaceutical compounds due to their multifaceted roles as solvents, catalysts, and reagents. Integrating of NaDES into synthesis protocols represents a broader effort to rethink and optimise the entire synthesis process, in line with global initiatives to enhance sustainability of chemical production.

### NaDES in extraction

**Fig. 19:**
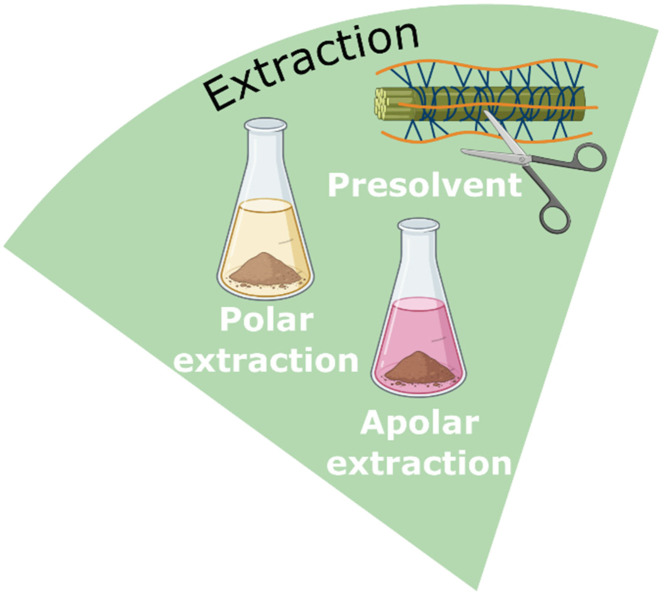
NaDES applications in the extraction of pharmaceutical compounds.

In the pharmaceutical industry today, the choice of solvent for compound extraction depends on the specific nature of the target compounds. Polar compounds are primarily extracted using aqueous or alcoholic solvents, while non-polar molecules are extracted with organic solvents such as hexane, DCM, toluene, dimethyl carbonate, and ethyl acetate. The broad range of polarities offered by NaDES makes them versatile for the extraction of both polar and non-polar compounds. NaDES have been successfully applied in the extraction of various metabolites from plant, animal, fungal, and bacterial biomass.^80–83^ Their ability to form hydrogen bonds enhances the extraction of more rigid and difficult-to-access biomass, such as wood and bark (Fig. 19).

#### NaDES as pre-treatment solvent

**Fig. 20:**
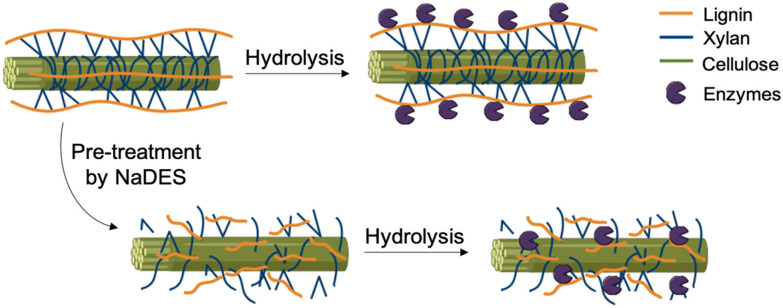
Pretreatment with NaDES to improve subsequent enzymatic hydrolysis.

Pre-treatment is often applied to facilitate solvent extraction, particularly in the processing of lignocellulosic biomass for improved biofuel production (Fig. 20).^28^ Lignocellulosic pretreatments can be either physical or chemical. Steam explosion, a commonly used physical process, is energy-intensive due to the high temperatures and pressures required. Chemical dissolution of lignin, in contrast, typically involves harsh acidic or alkaline conditions. Some ILs and NaDES have demonstrated the ability to dissolve lignin, offering the potential for pretreatments under milder conditions. NaDES, in particular, are becoming increasingly attractive compared to ILs due to their lower cost and reduced environmental impact.^28^

Procentese *et al.*^84^ studied the efficacy of ChCl/glycerol (1 : 2) in the pretreatment of agro-industrial food waste, such as apple residues, potato peels, coffee skins, and brewery spent grains, for the production of fermentable sugars. This pretreatment with ChCl/glycerol was compared to conventional methods like steam explosion and alkaline dissolution. The pre-treated biomass was subsequently enzymatically digested to produce fermentable sugars. The study demonstrated that the sugar yields and lignin removal achieved through eutectic solvent pretreatment are comparable to, or even exceed, those obtained by traditional pretreatment methods.

By serving as a pretreatment solvent, NaDES enable rapid access to the metabolites present in biomass, thereby reducing the number of steps required for processing.

#### NaDES as extraction solvent for polar compounds

Depending on their nature, polar metabolites may exhibit optimal solubility in alcohols such as ethanol or methanol, in water, or in hydroalcoholic mixtures. Due to their ability to form hydrogen bonds with metabolites, NaDES can dissolve a wide range of polar compounds. Furthermore, the polarity of eutectic mixtures can be adjusted according to the eutectic partners involved, their respective ratios, and the potential addition of a third component, such as water.^39^ The selection of the most suitable NaDES for extracting specific metabolites can be guided by modelling software and then validated through laboratory testing.

For instance, Tzani *et al.*^85^ conducted a comparative study of different NaDES in the extraction of metabolites with antioxidant potential from ginger. The mixture of betaine, lactic acid, and water (1 : 2 : 2.5) yielded the highest amount of total phenolic compounds and exhibited the best antioxidant activity, outperforming the betaine/glycerol (1 : 3) and glucose/lactic acid/water (1 : 5 : 6.2) mixtures. The optimal NaDES also demonstrated superiority over conventional extraction solvents, such as ethanol, water, and ethanol/water (70 : 30 v/v). In a subsequent study on the valorisation of phenolic compounds from Greek propolis, Tzani *et al.*^86^ highlighted the significant role of water content in NaDES on extraction efficiency. They predicted the optimal ratio for extracting the target metabolites and conducted tests to validate their findings.

NaDES are often used not only for extraction but also for stabilising metabolites, making them valuable excipients. Retaining the eutectic solvent used for extraction as a stabiliser in the final formulation can eliminate the need for isolation and purification steps. Punzo *et al.*^80^ emphasised the value of this strategy in recovering biowaste from wine production. The extract obtained from grape pomace using the NaDES betaine/citric acid (1 : 1) contains mainly polyphenols, in particular malvidin. The NaDES formulation enhances the skin bioavailability of malvidin, resulting in a cosmetic product with good antioxidant and anti-inflammatory activity.

The extract obtained with NaDES can also be considered for oral administration formulations. da Silva *et al.*^87^ focused on the phenolic compounds in blueberries, which exhibit promising activities in inhibiting cell proliferation, preventing obesity, type 2 diabetes, inflammation, and cardiovascular disease, but the use of which is limited by low oral bioavailability. The authors used ChCl/glycerol/citric acid/water (0.5 : 2 : 0.5 : 0.75) as an extraction solvent and oral absorption vehicle for these metabolites, particularly anthocyanins. The ready-to-use NaDES-based extract increased the bioavailability of anthocyanins by 140% compared to the organic solvent extract. This promising extract was then tested in *in vitro* and *in vivo* gastrointestinal digestion assays in rats, demonstrating increased intestinal stability and bioavailability of phenolic compounds. NaDES delayed the neutralisation of anthocyanins by gastric fluid.

#### NaDES as extraction solvent for apolar compounds

The recent development of apolar NaDES has further expanded their solubilisation range. Apolar NaDES can be a combination of several fatty acids, a fatty acid with a monoterpene or a quaternary ammonium, multiple monoterpenes, or a monoterpene with an organic acid.^1,38,42,45,88^ Wils *et al.*^89^ highlighted the effectiveness of various apolar NaDES for extracting lipids and pigments from the microalga *Arthrospira platensis*, commonly known as spirulina. NaDES composed of a monoterpene and an organic or fatty acid, such as menthol/lactic acid (1 : 2), menthol/levulinic acid (1 : 2), and menthol/octanoic acid (1 : 1), demonstrated selectivity for free fatty acids (FFA) and carotenoids, with extraction performances comparable to those of dimethyl carbonate and ethyl acetate. NaDES composed solely of fatty acids, such as nonanoic acid/lauric acid (3 : 1) and nonanoic acid/decanoic acid/lauric acid (3 : 2 : 1), exhibited similar selectivity while achieving extraction yields twice as high as those of conventional solvents. Additionally, the apolar NaDES studied had the advantage of low viscosity.

Apolar extractions are often combined with polar extractions. Extractions can be performed sequentially, beginning with a polar NaDES extraction followed by an apolar NaDES extraction. Alternatively, a biphasic system can be used, involving the simultaneous application of polar and apolar NaDES to form an immiscible system. In their study on the recovery of apolar compounds from spirulina, Hilali *et al.*^90^ demonstrated the superiority of the biphasic method in terms of process efficiency and yield. They described a straightforward ultrasonic extraction protocol in which biomass is mixed with apolar NaDES menthol/1,2-octanediol (1 : 1) and polar NaDES glycerol/glucose (2 : 1). The extraction yields were four times higher for chlorophylls and free fatty acids and twice as high for carotenoids. Additionally, the biphasic method enhanced the purification of the phycocyanin-enriched polar fraction. Vieira *et al.*^42^ reported a similar protocol for extracting compounds with high antioxidant activity from rosemary. In their study, a menthol/lauric acid mixture (2 : 1) efficiently extracted carnosic acid and carnosol, whereas rosmarinic acid showed a preference for the lactic acid/glucose mixture (5 : 1). Furthermore, the extracted antioxidant metabolites exhibited greater stability in NaDES than in methanol.

As with polar metabolites, NaDES can be used both as extraction solvents and as excipients for apolar compounds. Jamaleddine *et al.*^91^ employed this approach to valorise metabolites from tomato pomace. They developed four eutectic mixtures with varying polarities: the menthol/lactic acid mixture (8 : 1) enabled the extraction of lipophilic compounds such as lipids, carotenoids, and tocopherols; glycerol/proline (1 : 2.5) was effective for flavonoids; glycerol/glucose (3 : 1) was used to recover flavonoids, tannins, and phenolic compounds; and finally, glucose/lactic acid (5 : 1) was used to extract phenolic compounds. Various cosmetic products were formulated from these extracts, including a lip balm, an exfoliating mask, a water-soluble mask, and a moisturising cream. In these formulations, NaDES were used not only as excipients to solubilise active ingredients but also, depending on their properties, as moisturising, humectant, and pH-adjusting agents.

NaDES demonstrate extraction performance for both polar and apolar compounds that equals or exceeds that of conventional solvents. Their strong extracting power is based on two main mechanisms: direct interaction with the targeted compounds, often *via* hydrogen bonds, and their action as a pre-treatment solvent, which disrupts the cell wall and releases compounds from the plant matrix. Innovative approaches involving biphasic systems have been developed to enable the simultaneous extraction of polar and apolar compounds. Furthermore, most NaDES are non-volatile and possess characteristics similar to excipients, allowing the direct incorporation of extracts into formulations without the need for isolation and purification steps.

### NaDES as excipient

**Fig. 21:**
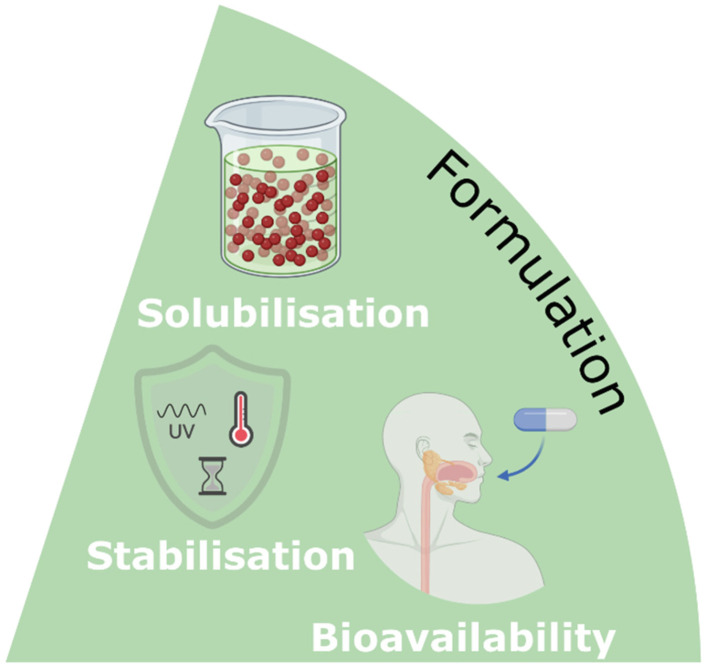
NaDES applications in pharmaceutical formulations.

NaDES are excellent candidates for excipients in improving the solubility, stability, and bioavailability of various formulations, owing to their ability to establish hydrogen bonds and van der Waals interactions with active ingredients (Fig. 21).

#### NaDES as solubilising agent

The low solubility of certain active ingredients in water limits their oral bioavailability. Unlike water, hydrophilic NaDES have the ability to solubilise lipophilic compounds.^30^ The addition of excipients such as NaDES, which interact non-covalently with active ingredients, can significantly enhance their solubility.

Lu *et al.*^92^ evaluated various NaDES for their ability to solubilise non-steroidal anti-inflammatory drugs (NSAID), including aspirin, paracetamol, ibuprofen, ketoprofen, and naproxen. They found that ibuprofen was 5470 times more soluble in the NaDES composed of ChCl/levulinic acid (1 : 2) than in water. In comparison, the solubility of ibuprofen in a menthol/camphor (1 : 1) mixture was only increased by a factor of four, highlighting the importance of thorough screening when selecting the most effective alternative solvent. Additionally, the introduction of a third eutectic partner can further improve solubility, as observed with ketoprofen, whose solubility increased 960-fold in the eutectic mixture of ChCl/glycolic acid/oxalic acid (1 : 1.6 : 0.4).

Similarly, Li and Lee^93^ demonstrated the enhanced solubilisation of antifungals such as itraconazole and posaconazole using NaDES. Posaconazole was found to be 8840 times more soluble in a eutectic mixture of ChCl and glycolic acid (1 : 2) than in water, while itraconazole's solubility increased by up to 53 600 times in the ChCl/glycolic acid/oxalic acid mixture (1 : 1.7 : 0.3).

NaDES are also excellent solubilisers for macromolecules such as proteins, starch, and DNA. Dai *et al.*^25^ showed that DNA and gluten proteins were respectively 35 and 101 times more soluble in a tertiary mixture of lactic acid/glucose/water (5 : 1 : 3) than in pure water. These findings underscore the potential importance of NaDES in biological systems and open up promising prospects for the formulation of protein- and DNA-based drugs. Therapeutic proteins, such as insulin, growth hormone, and monoclonal antibodies, are used in the treatment of conditions such as diabetes, growth hormone deficiency, chronic inflammatory diseases, cancer, and transplant rejection.^94^ DNA, on the other hand, is utilised in gene therapy for the treatment of monogenic diseases and certain cancers.^95^

Apolar NaDES have been employed as solubilising agents in formulations for topical applications. Al-Akayleh *et al.*^96^ developed a NaDES based on capric acid/menthol (4 : 1) to solubilise fluconazole and mometasone, antifungal and anti-allergy drugs, respectively. The system successfully enhances drug solubility and does not cause skin oedema or inflammation after topical application.

#### NaDES as stabilising agents

Many active ingredients are unstable in aqueous media and can react with water. This is particularly true for esters such as aspirin, which hydrolyses into salicylic acid and acetic acid. Due to its instability, aspirin is not marketed in an aqueous formulation, as it has a half-life of less than two weeks even at optimal pH. Lu *et al.*^92^ observed an increase in aspirin's stability when dissolved in a eutectic mixture of ChCl/propanediol (1 : 2). The non-covalent interactions provided by NaDES present a promising alternative for the liquid oral formulation of aspirin.

Similarly, Olivares *et al.*^97^ demonstrated enhanced stability of the β-lactam antibiotic imipenem, combined with clavulanic acid, when dissolved in a betaine/urea mixture (1 : 1.5) compared to an aqueous solution. β-Lactams are known for their physicochemical instability in aqueous media, as the opening of the four-membered ring leads to a loss of antimicrobial activity. The betaine/urea mixture (1 : 1.5) formulation effectively preserves the activity of imipenem combined with clavulanic acid. This new formulation is particularly interesting for continuous injectable administration.

NaDES could also play a role in preserving therapeutic proteins. Due to their thermosensitivity, protein drugs face numerous challenges, including the need to maintain the cold chain during shipment, storage, and handling. Daneshjou *et al.*^98^ examined the stability of ABCI chondroitinase, an enzyme used in the treatment of spinal injuries, in aqueous NaDES solutions based on ChCl/glycerol (1 : 2) and betaine/glycerol (1 : 2). Their study revealed that the enzymatic activity of the enzyme could be maintained at over 95% for fifteen days at −20 °C in the presence of NaDES, whereas it was lost after just five days in aqueous phosphate buffer. Furthermore, at 4 °C, the use of NaDES increased stability by a factor of 6.5 compared to conventional storage methods.

Lee *et al.*^99^ conducted a similar study involving human interferon-α2 (IFN-α2), a protein drug used to treat hepatitis B and C as well as leukemia. The ChCl/fructose mixture (1 : 1) demonstrated improved stability of IFN-α2 over both short (two hours) and long-term (three months) storage periods at elevated temperatures of 37 °C, 50 °C, and 70 °C compared to solubilisation in phosphate buffer. Structural analyses confirmed that the protein retained its integrity and activity when stored long-term in NaDES at 37 °C.

#### NaDES as bioavailability promoter

Bioavailability refers to the amount of an active ingredient that reaches the bloodstream. As the oral route is the most convenient for systemic application, low oral bioavailability is a significant limiting factor for the use of certain active molecules.^100^ Faggian *et al.*^101^ conducted a proof-of-concept study on rutin solubilised in a eutectic mixture of proline/glutamic acid (2 : 1). They observed an increase in the area under the curve (AUC) and maximum blood concentration of rutin when using NaDES compared to aqueous solvents. This strategy can be adapted to more lipophilic active ingredients, which are often poorly absorbed, undergo significant intestinal and hepatic first-pass metabolism, and are subject to hepatobiliary excretion and efflux by systems such as P-glycoprotein.

Sut *et al.*^102^ applied this approach to improve the bioavailability of berberine, a metabolite found in the roots, rhizomes, and stems of species like *Berberis* spp., *Coptis* spp., and *Hydrastis* spp., commonly used in traditional Chinese medicine. Despite its many pharmacological activities, berberine is poorly bioavailable when administered orally due to poor absorption and extensive metabolism. Sut *et al.* demonstrated the effectiveness of NaDES composed of proline/lactic acid/malic acid/water (1 : 0.2 : 0.3 : 0.5) in enhancing the solubility and bioavailability of berberine.

A similar strategy was proposed by Jeliński *et al.*^103^ for curcumin, a compound that is poorly soluble in water, has low bioavailability, and is easily degraded in the presence of light. Jeliński *et al.* used a eutectic system based on ChCl/glycerol (1 : 1) to improve curcumin extraction from pulverised turmeric, as well as to enhance its solubility, bioavailability, and photostability.

NaDES are not only excellent excipients for oral administration but also for transdermal delivery, especially with the recent development of lipophilic NaDES. These systems often involve combinations of fatty acids but can also include more polar compounds such as sugars or polyalcohols. Wils *et al.*^104^ explored the extraction of fatty acids and pigments from spirulina using NaDES, as well as potential topical formulations derived from these extracts. They utilised apolar NaDES consisting of nonanoic acid/lauric acid (3 : 1) and polar NaDES consisting of glucose/glycerol/water (1 : 2 : 4). The extracts obtained exhibited anti-staphylococcal bactericidal properties, suggesting their potential use as preservatives. Given their non-toxicity towards keratinocytes, these extracts could be suitable for topical formulations.

NaDES are biosourced excipients generally available in large quantities. They enhance the solubility and stability of active ingredients and can be used in both topical and systemic applications. In systemic applications, NaDES could enable the use of certain active ingredients previously limited by low bioavailability. Studies have shown improved bioavailability of active ingredients *via* oral routes, with injectable and transdermal routes also being considered.

### NaDES as active ingredients (TheDES)

**Fig. 22:**
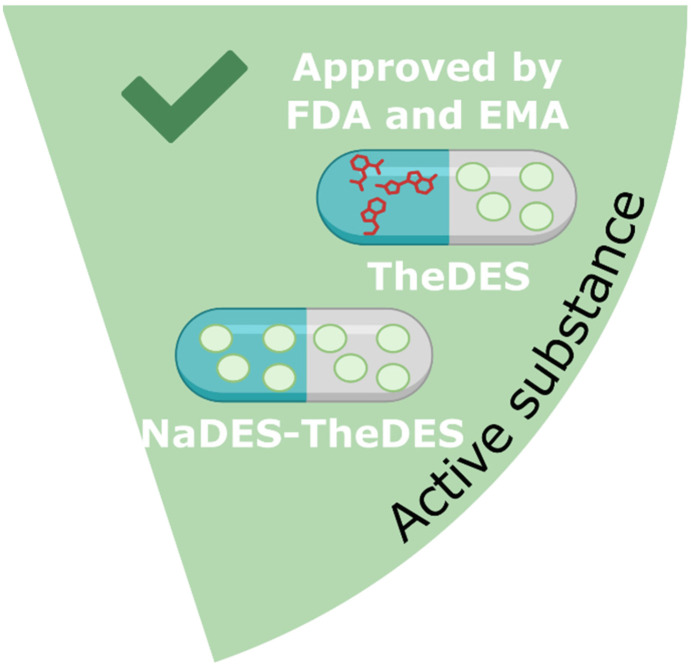
Applications of NaDES as active ingredients.

In the presence of specific compounds and in defined proportions, certain active ingredients can behave as eutectic compounds. This type of mixture is known as a therapeutic deep eutectic solvent (TheDES, Fig. 22).^1^

#### TheDES composed of several active ingredients

Currently, the only TheDES on the market, approved by the FDA and the EMA, are local anaesthetic mixtures derived from cocaine.^1^ Four formulations are available on the market. The first, approved in 1992 by the FDA, is EMLA® cream, composed of a 1 : 1 mixture of lidocaine and prilocaine, used for local anaesthesia. Today, EMLA® is also available in patch form. Subsequently, two lidocaine/tetracaine (1 : 1) based local anaesthetics were introduced as SYNERA® (patch) and PLIAGLIS® (cream). Since 2019, FORTACIN®, a lidocaine/prilocaine (1 : 1) spray, has been indicated for the management of primary premature ejaculation.^1^

Depending on their functional groups, active ingredients tend to act as either HBA or HBD. Due to the protonation of its tertiary amine group under physiological conditions, lidocaine behaves similarly to quaternary ammoniums, such as ChCl or betaine.^105^ It acts as a HBA and can bind to various HBD. The active ingredients most studied for their HBD capacity are carboxylic acids, and occasionally sulfonic acids. This is the case for many NSAID as well as for some emollients like azelaic acid and docusate. Formulations combining lidocaine and NSAID have been developed for use in dressings or as local anti-inflammatory treatments, such as for mucositis. Additionally, combining lidocaine with emollients like docusate generally enhances skin permeability.^105^

The eutectic phenomenon allows solid active ingredients to become liquid at room temperature, simplifying their formulation and facilitating absorption by eliminating the dissolution stage. For example, mixing lidocaine and prilocaine in equimolar proportions reduces their respective melting points from 68 °C and 37 °C to 18 °C.^106^ Lidocaine can also form eutectic mixtures with natural small molecules such as menthol or thymol. This effect is also observed with other active ingredients and is discussed in the following section.

#### TheDES consisting of an active ingredient combined with a natural product

The choice of eutectic partner depends not only on the polarity of the active ingredient but also on the interactions it can induce. Table 6 presents the melting temperatures of various TheDES compared with those of their respective components.^41^

**Table 6:** TheDES melting temperatures compared with those of their respective components^41^

TheDES compound 1/compound 2 (ratio)	NaDES melting temperature (°C)	Melting temperature of compound 1 (°C)	Melting temperature of compound 2 (°C)
Propranolol/capric acid (3.5 : 6.5)	15.4	96	31.9
Ibuprofen/dl-menthol (2.5 : 7.5)	13	75–77	39
Ibuprofen/l-menthol (3 : 7)	19	75–77	39
Ibuprofen/1,8-cineole (2 : 3)	−13	75–77	1.5
Lidocaine/lauric acid	6	68	43.8
Itraconazole/phenol (2,4 : 7,6)	<0	166.2	40.9
Resorcinol/ChCl (1 : 1)	6	109.8	305
Aspirin/ChCl (1 : 1)	Room temperature	135	305
Ascorbic acid/ChCl (1 : 2)	Room temperature	190–192	305
Coenzyme Q10/l-menthol (6 : 4)	37	50–52	39

**Fig. 23:**
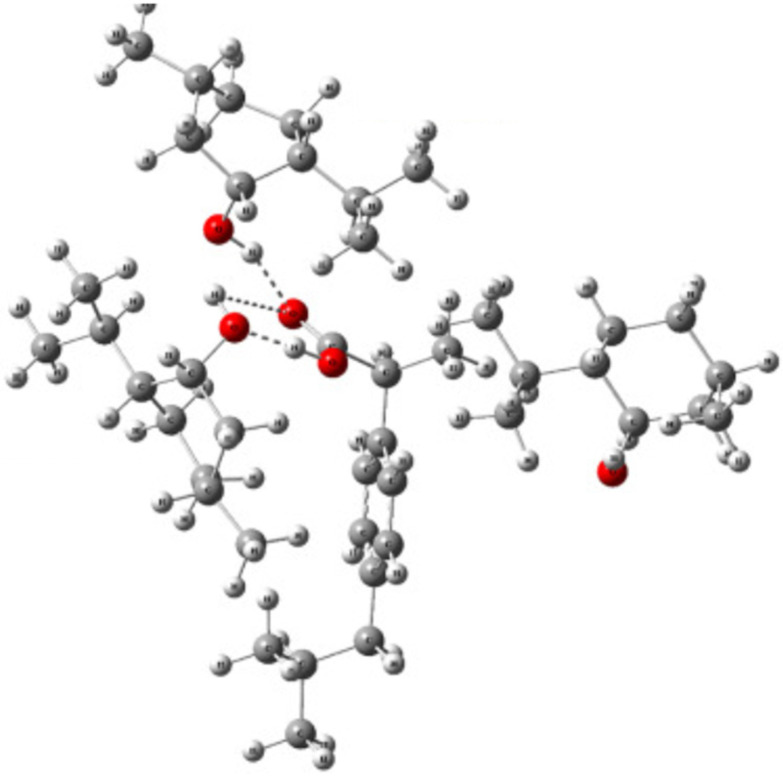
Hydrogen interactions involved in the ibuprofen/menthol (1 : 3) mixture. This figure has been adapted from ref. 41 with permission from Elsevier, copyright 2021.

Menthol, a monoterpene commonly found in peppermint essential oil, is widely used in TheDES due to its low toxicity, ease of access, and biocompatibility with many eutectic partners.^96,107^ It is also considered a permeability enhancer for dermal administration. Menthol is often combined with various active ingredients such as ibuprofen, aspirin, or coenzyme Q10.^40^ As shown in Fig. 23, its hydroxyl group promotes the formation of hydrogen bonds with the carboxyl or carboxylic acid groups of partner molecules. Additionally, the stereochemistry of menthol influences its eutectic behaviour, altering its ratio and eutectic point (as shown in Table 6).^41^

Fatty acids have proven to be effective partners in the formation of eutectic mixtures with active ingredients. For example, capric acid, a saturated fatty acid with a C_10_ carbon chain, has been combined with propranolol and risperidone to improve their bioavailability.^108,109^ Propranolol, a β-blocker commonly prescribed for hypertension, and risperidone, an antipsychotic widely used as a first-line treatment for schizophrenia and bipolar disorder, both suffer from limited oral bioavailability due to a significant hepatic first-pass effect. Alternative routes of administration are being explored to bypass the liver and deliver these drugs directly into the systemic bloodstream. The transdermal route is particularly promising due to its non-invasive nature and ease of administration.^108^ By forming a eutectic mixture with these active ingredients, capric acid facilitates skin application and enhances transdermal penetration.^109^

Organic acids can also be used to form TheDES containing more polar active ingredients, such as nitroimidazole antibiotics. Metronidazole, often prescribed as a first-line treatment for bacterial vaginosis, is typically administered as oral tablets over a seven-day course. However, this approach has drawbacks, including poor patient compliance, adverse effects, and unnecessary systemic exposure to an antibiotic, which can contribute to the emergence of resistance. To address these issues, Li *et al.*^110^ developed a TheDES based on a 1 : 1 mixture of metronidazole and maleic acid, incorporated into a solid vaginal insert consisting of a polycaprolactone matrix. This formulation provides controlled drug release over a seven-day period, offering an alternative to oral treatment.

#### TheDES whose active ingredient is a natural product

Natural products can serve not only as eutectic partners but also as active ingredients. Some PRIM or HEVO exhibit therapeutic effects, which can sometimes be amplified by synergy when combined with other metabolites within a NaDES.

Santos *et al.*^111^ have been working on improving the bioavailability of anti-tuberculosis treatments to minimise adverse effects and prevent the emergence of antibiotic resistance resulting from the administration of high doses. They combined treatment with ethambutol, a bacteriostatic agent used against the *Mycobacterium tuberculosis* complex, with an arginine-based treatment, which has been reported to reduce tuberculosis symptoms in HIV-uninfected patients. To achieve this, they formulated a TheDES-NaDES based on citric acid/arginine/water (1 : 1 : 7) in combination with ethambutol, also formulated in a TheDES. These eutectic mixtures improve the solubility of ethambutol, positioning the TheDES-NaDES as a promising candidate for a new anti-tuberculosis therapy.

The incorporation of limonene into a TheDES has been investigated for its potential use as an anti-cancer agent. Although limonene possesses anti-proliferative activity, its toxicity to healthy cells limits its widespread use. To address this limitation, Pereira *et al.*^112^ developed several TheDES formulations, including menthol/limonene (1 : 1), capric acid/limonene (1 : 1), and ibuprofen/limonene (1 : 4) mixtures. According to the data obtained, all these formulations display anti-tumor properties, but only the ibuprofen/limonene (1 : 4) combination was able to inhibit the proliferation of HT29 lineage cells without affecting the viability of healthy cells. These results suggest that the mechanism of action of the limonene/ibuprofen (1 : 4) mixture differs from that of ibuprofen and limonene taken in isolation, implying a synergistic effect of TheDES.

Mano *et al.*^113^ have developed a rapid-release oral delivery system for an antibacterial agent. They combined a TheDES based on ChCl/mandelic acid (1 : 2) with a gelatin polymer. The formulation was obtained by electrospinning the gelatin and TheDES to produce nanofibers. This system enables the rapid dissolution of the active ingredient through the degradation of the polymer in the mouth, without the need for water. The nanofibers demonstrated an antibacterial effect against both Gram-positive and Gram-negative bacteria, with no observed toxic effect *in vivo*. However, further analysis of their pharmacokinetic properties is needed to establish their added value compared to traditional drugs.

Similarly, Silva *et al.*^114^ developed a controlled delivery system for a TheDES. They first designed a new TheDES based on ascorbic acid/ChCl (1 : 2) and demonstrated that the antioxidant activity of ascorbic acid was preserved for up to six months in this form. They also demonstrated the effectiveness of this TheDES in solubilising dexamethasone, a steroidal anti-inflammatory drug. After determining the half-maximal effective concentration (EC_50_ = 1.49 mg mL^−1^) of the TheDES containing dexamethasone, they incorporated it into a starch and poly-ε-caprolactone matrix to create a controlled drug delivery system.

Exploiting the eutectic nature of an active ingredient in formulation facilitates its solubilisation and reduces the need for multiple excipients. As a result, this approach minimises the number of compounds involved in drug manufacturing. Additionally, TheDES-NaDES can play a crucial role in novel drug delivery approaches, as illustrated in the previous examples. This aspect will be explored in greater detail in the next section.

### NaDES as a tool in innovative synthesis and formulation methods

**Fig. 24:**
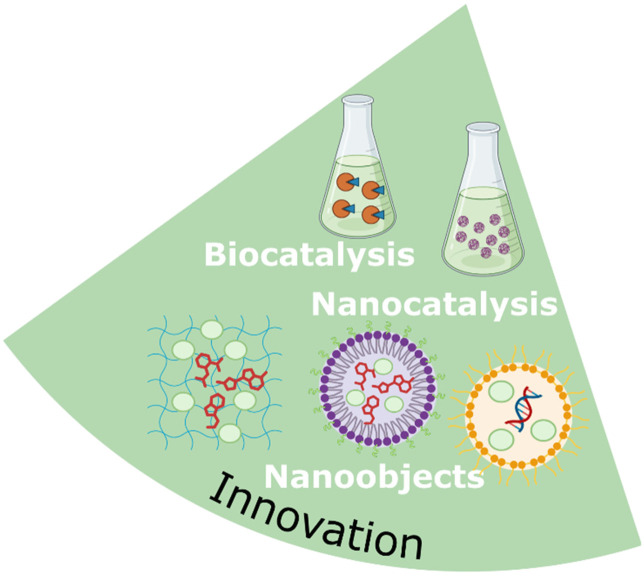
NaDES applications in innovative strategies.

The pharmaceutical industry today faces significant challenges in both drug synthesis and formulation. In drug synthesis, it is crucial to find sustainable alternatives to petroleum-derived solvents and dwindling supplies of metal catalysts. In formulation, the focus is on facilitating drug administration and controlling distribution to minimise side effects and treatment constraints, ultimately improving patients’ quality of life. To address these challenges, nanotechnology and biotechnology are becoming increasingly important in the pharmaceutical sector (Fig. 24). Nanotechnology allows for the exploration and manipulation of phenomena at the nanometric scale, where materials exhibit unique properties. In contrast, biotechnology leverages living organisms—sometimes genetically modified—and their components, such as enzymes or pigments.

#### NaDES in innovative synthesis strategies

The biocatalysis field has recently realised the potential of NaDES especially as solvent.^115^ While most enzymes are soluble in aqueous conditions, their substrate has limited solubility in water, resulting in low reaction yields. To avoid this problem, NaDES can be used as solvent or co-solvent to increase the solubility of the substrate.

For example, ChCl-based NaDES have been used as co-solvents in biocatalysis with whole cells to boost the production of vanillin, a precursor for active ingredients.^116^ The reaction, catalysed by *Lysinibacillus fusiformis* CGMCC1347 cells from isoeugenol, saw production yields increase by up to 142% with the incorporation of relatively small amounts of ChCl-based NaDES (1% v/v in water) compared to yields obtained in pure water. Subsequently, the authors immobilised *Lysinibacillus fusiformis* CGMCC1347 cells in PVA-alginate beads, allowing the catalyst to be reused while maintaining catalytic activity over at least thirteen cycles, ultimately increasing yields by up to 181%.

**Fig. 25:**
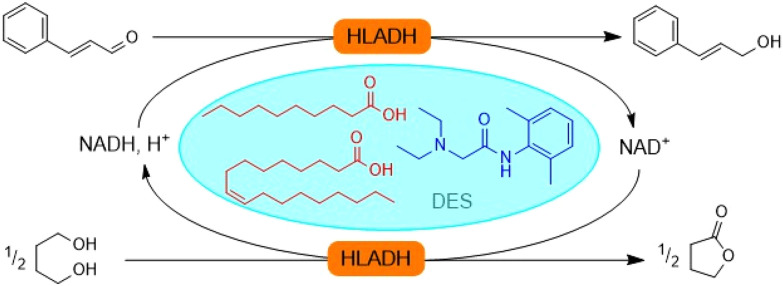
Reduction of cinnamaldehyde catalysed by HLADH coupled with oxidation of 1,4-butanediol for cofactor regeneration in a DES composed either of lidocaine/oleic acid (1 : 1) or lidocaine/decanoic acid (1 : 2).

In a recent study, Zhang *et al.*^88^ demonstrated the potential of lidocaine-based DES to facilitate the biocatalytic reduction of hydrophobic aldehydes, such as cinnamaldehyde (Fig. 25). The hydrophobic nature of the DES, either lidocaine/oleic acid (1 : 1) or lidocaine/decanoic acid (1 : 2), enabled an increased substrate loading, reaching industrially relevant concentrations (100 g L^−1^) for cinnamaldehyde. An optimal aqueous buffer content of 20% within the DES maintained a homogeneous single-phase system while ensuring sufficient enzyme hydration. The reaction, catalysed by horse liver alcohol dehydrogenase (HLADH), outperformed the conventional buffer system (Tris-HCl, 50 mM, pH 7.5) by a factor of three. With a PMI of 1.5, this biocatalytic approach surpassed the organometallic reduction of cinnamaldehyde by a factor of 100.^117^ In the chemical synthesis, 2-propanol was used both as a solvent and reactant, achieving a relatively high RME of 80%. However, this method required a large quantity of zirconium-based transition metal catalyst grafted onto silica, with no consideration of catalyst recovery. In contrast, the biocatalytic reduction was coupled with the oxidation of 1,4-butanediol to regenerate NAD^+^, offering several advantages: a higher yield (90% compared with 79% for the organometallic route), operation under milder conditions, and improved sustainability.

Nanotechnologies, particularly catalytic nanoparticles, are widely used to enhance performance in chemical synthesis. Working at the nanoscale allows for an increase in the contact surface area of a catalyst, which promotes interaction with the substrate and leads to improved efficiency.^40^ The contact surface is closely related to the size and morphology of the nanoparticles. Therefore, it is crucial to control these parameters during nanoparticle synthesis and to ensure their post-synthesis stability, as they tend to agglomerate. The choice of solvent for synthesis and storage is thus of great importance. Due to their dispersive properties, thermal stability, ionic conductivity, and wide electrochemical window, NaDES offer precise control over nanoparticle size and morphology while preventing agglomeration.

Lu *et al.*^118^ utilised a eutectic mixture of citric acid and dimethylurea (1 : 1.5) for the synthesis of copper iron oxide nanoparticles (CuFeO_2_), which were subsequently used in the preparation of imidazo[1,2-*a*]pyridine. Imidazo[1,2-*a*]pyridine derivatives are of significant synthetic interest due to their diverse pharmacological and therapeutic properties, including antiviral, antitubercular, and antiepileptic activities.

The derivatives were synthesised *via* a one-pot, three-component reaction involving 2-aminopyridines, aldehydes, and alkynes, conducted in a NaDES system containing catalytic nanoparticles. The nanoparticles exhibited excellent efficiency and stability, with reaction yields remaining above 90% over six cycles.

A similar three-component reaction using related substrates was previously patented in methanol with excess acetic acid as a reagent.^119^ To compare the NaDES method with the patent, specific substrates from the ‘reaction scope’ section were used. Although both approaches gave comparable yields (∼85%), the patented method demonstrated a superior RME of 84%, as the reactants were employed in equimolar quantities. In both cases, the primary contributor to PMI was the solvent—either methanol or NaDES. Lu *et al.* demonstrated that NaDES could be reused over six cycles with only a 5% decrease in yield. Accounting for solvent recycling in the calculations, the PMI was reduced from 9.2 to 2.9, outperforming the patented method, which had a PMI of 7.6.

Similarly, Oh and Lee^120^ synthesised gold nanoparticles in a eutectic solvent composed of ChCl and malonic acid (1 : 1), which were then used for catalytic and diagnostic purposes. Using the NaDES as both reaction medium and structuring agent, the size and structure of the nanoparticules were found to be homogeneous. This approach is noteworthy as it requires less gold for equivalent catalytic activity, thanks to the increased contact surface. The nanoparticules were found to catalyse well the reduction of 4-nitrophenol to 4-aminophenol and to detect DNA targets as colourimetric probes.

#### NaDES in innovative formulation strategies

Nanotechnologies are also widely used in drug formulation. Many active ingredients are formulated as nanocrystals or encapsulated in polymeric nanoparticles.^1^ As with catalytic nanoparticles, the size and morphology of these particles are crucial for their absorption and distribution in the body. NaDES are employed as a synthesis medium and excipient for storing nanoparticles and nanocrystals.

Li *et al.*^121^ developed a transdermal formulation using mesoporous silica nanoparticles impregnated in NaDES hydrogels for the topical treatment of rheumatoid arthritis. Nanoparticles containing methotrexate and nanoceria were first prepared in a NaDES composed of arginine/citric acid (3 : 1), then incorporated into a carbomer hydrogel. The hydrogel/NaDES system has a high affinity for the skin, enabling enhanced penetration of the active ingredients.

Beyond serving as reaction media, NaDES can also be used in the synthesis of nanoobjects. Cecone *et al.*^122^ explored the use of a ChCl/citric acid mixture at different ratios as both solvent and cross-linking agent in the synthesis of cyclodextrin-based nanosponges. Interestingly, depending on the ratio of ChCl to citric acid, nanosponges show positive charge. β-Cyclodextrin-based polymers are commonly used to control drug release and adsorb undesirable substances in various sectors, including pharmaceuticals, food, and environmental applications. The use of NaDES eliminates the need for organic solvents typically required in the production of these polymers.

**Fig. 26:**
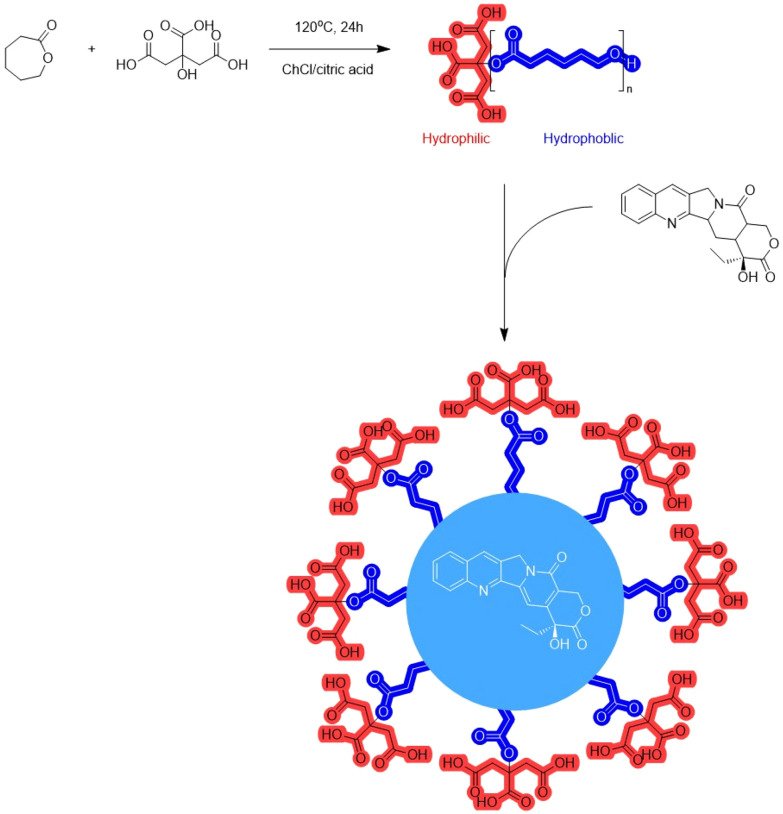
Synthesis of poly(e-cp-*co*-CA) nanomicelles from citric acid and caprolactone.

Pradeepkumar *et al.*^123^ exploited the dual role of NaDES in the preparation of a nanomicelle composed of ε-caprolactone-citric acid (ε-cp-*co*-CA), enabling the controlled release of anticancer agents (Fig. 26). In this approach, the ChCl/citric acid (1 : 2) mixture acts as both solvent and citric acid source for caprolactone functionalisation. The efficacy of the ε-cp-*co*-CA vector was evaluated by encapsulating camptothecin, a topoisomerase I inhibitor isolated from *Camptotheca acuminata* bark. The controlled release of camptothecin was monitored using the MTT cell viability assay and was found to be time-dependent. This suggests that camptothecin-loaded poly(ε-cp-*co*-CA) nanomicelles could represent a promising system for antitumor treatments.

NaDES could also find applications in the field of DNA nanotechnology. The production of these nanotechnologies is complex, often requiring chemical reactions or processes to be conducted under strictly anhydrous conditions. However, nucleic acid processing is typically carried out in aqueous media due to the lack of suitable solvents. Zhao *et al.*^124^ investigated the use of NaDES as an anhydrous medium for guanine-rich DNA sequences that fold into G-quadruplex helices. They found that the G-quadruplex structure is more stable in NaDES than water and can survive above 110 °C. This advancement could significantly enhance the application of DNA nanotechnologies in fields such as supramolecular chemistry, medicinal chemistry, and nanoscience.

NaDES contribute to the advancement of innovative technologies such as nanotechnology and biotechnology. Their ability to generate hydrogen bonds helps ensure the stability and efficacy of nanoparticles and biocatalysts. Through this, NaDES research is driving the development of novel synthesis and formulation strategies in the pharmaceutical industry.

## Limits to the use of NaDES in the pharmaceutical industry

### Overcoming NaDES issues

One of the main disadvantages of deep eutectic mixtures compared to conventional solvents is their high viscosity, which is most likely caused by the extensive network of hydrogen bonds and van der Waals interactions.^50^ This characteristic is particularly problematic when NaDES are used as solvents. As extraction solvents, their high viscosity can reduce the diffusion coefficients of metabolites, leading to longer extraction times and potentially altering the solubility of target compounds.^18^ In organic synthesis, high viscosity can impede mass and energy transfer during chemical reactions, thereby affecting reaction yields.^46^

To overcome these challenges, the addition of water or glycerol and gentle heating can be employed to disrupt the hydrogen bond network and weaken intermolecular forces while preserving the supramolecular structure of the solvent. As discussed earlier, a water content of 25% generally provides optimal stability. In their study on *Carthamus tinctorius* L., Dai *et al.*^63^ observed that this water content also resulted in the highest extraction efficiency for the polar metabolite carthamin. The addition of water may therefore be a viable strategy for extracting polar compounds. For less polar metabolites, apolar NaDES can be used, as they typically exhibit lower viscosity. Combining NaDES with physical techniques such as ultrasonic waves, microwave irradiation, or bead beating not only improves dispersion in the viscous solvent but also enhances mass transfer. Sed *et al.*^66^ reported increased yields in the extraction of proteins, carbohydrates, and lipids from microalgal biomass when using microwave-assisted or bead-beating methods. Similarly, Gu *et al.*^125^ demonstrated that ultrasonic waves significantly reduced the extraction time.

The upscaling of processes involving NaDES remains underexplored in the literature, with viscosity being one of the primary challenges in adapting NaDES for industrial applications. However, some companies have addressed this issue through technical innovations. For instance, GatteFossé has patented an extraction process employing sugar, polyol, and water mixtures—particularly a fructose/glycerol/water (1 : 1 : 5) blend—to extract active ingredients from plants such as *Aesculus hippocastanum*, *Withania somnifera*, and *Sechium edule*.^126^ This extraction is performed in a stirred reactor, followed by solid–liquid separation *via* centrifugation rather than filtration. The resulting extract is then filtered to 0.2 μm and directly incorporated into formulations. Additionally, GatteFossé has developed NaDES systems containing betaine in combination with glycol, polyol, or sugar for dissolving stilbenoids and their derivatives.^127^

However, due to their specific physicochemical properties and the limited availability of certain eutectic components, some NaDES are unsuitable for large-scale applications. In such cases, their implementation in flow-process microreactors for active ingredient synthesis or extraction could be an alternative.

NaDES can also serve as functional additives; for instance, in formulations, they can act as cosolvents to enhance the solubilisation of active ingredients or as preservatives and stabilisers, particularly in aqueous formulations.^92,93^ Moreover, some researchers have reported that small quantities of NaDES can improve the resolution of analytical methods for drug isolation. For example, Ramezani and Absalan^128^ utilised a eutectic mixture as a mobile phase additive in micellar liquid chromatography, enhancing the isolation of four key cardiovascular drugs.

While the non-volatile nature of most NaDES is generally associated with reduced toxicity, it can present challenges during the isolation and purification phases of synthesised or extracted substances. In traditional processes, organic solvents are typically separated from the compounds of interest by evaporation.^60^ For NaDES, chemists have developed alternative methods that often allow for their reuse. These methods may involve precipitation of the product, after adding water. In some cases, the excipient properties of NaDES justify their direct inclusion in a formulation, bypassing purification steps. However, it is essential to ensure the non-toxicity of the formulated NaDES.^18^

### Toxicity of NaDES

Although NaDES constituents are biosourced and often considered safe, it is important to note that a natural product is not inherently free of toxicity.^18^ Moreover, the toxicity of the final eutectic mixture may differ from that of its individual constituents.^129,130^

**Fig. 27:**
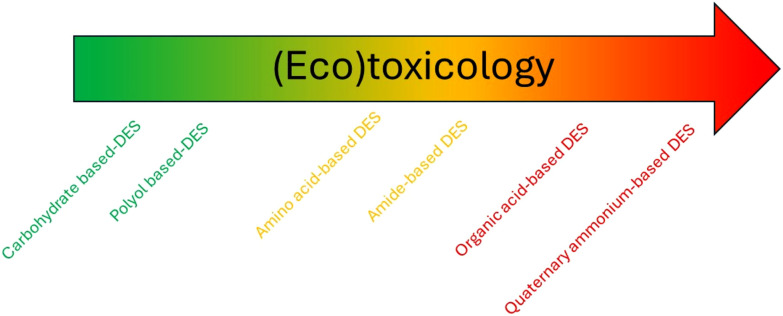
Classification of different types of DES according to their toxicity and eco-toxicity.

*In silico* studies have attempted to classify different types of NaDES according to their toxicity and ecotoxicity. For example, Halder and Cordeiro^131^ developed a quantitative structure–toxicity relationship (QSTR) model. Most *in silico* studies yield similar classifications, as shown in Fig. 27.^131–133^ However, some disparities between models exist, so these results should be interpreted with caution.

Similarly, *in vitro* results may vary depending on the methods used to assess toxicity and biodegradability. Most ecotoxicity assessments conducted on NaDES have relied on antibiograms. For example, Radošević *et al.*^130^ evaluated the antimicrobial effects of different NaDES on organisms such as *Escherichia coli*, *Proteus mirabilis*, *Salmonella typhimurium*, *Pseudomonas aeruginosa*, and *Staphylococcus aureus*. They tested a wide range of NaDES, including those based on quaternary ammonium, organic acids and sugars, as well as amino acids. Organic acid-based NaDES showed the strongest inhibition of bacterial growth, consistent with *in silico* studies. Moreover, this inhibition was greater than the effect of the acids alone, which may be explained by the synergistic effects within the NaDES.

Interestingly, De Morais *et al.*^129^ observed the opposite trend, where the acid alone had a greater effect than the NaDES mixture. They compared the ecotoxicity of ChCl and several organic acids, both alone and in eutectic mixtures, through *in vivo* tests on the marine bacterium *Vibrio fischeri* and measured the EC_50_. All the examined NaDES showed intermediate toxicity compared to the organic acids alone, with toxicity increasing with acid concentration. The discrepancy between these results can be attributed to the different methods used; Radošević *et al.* measured bacterial growth inhibition, while De Morais *et al.* assessed cytotoxicity. The antibiogram method used by Radošević *et al.* has limitations due to the high density and viscosity of many eutectic mixtures, and it is not suitable for continuously monitoring cell growth or changes in physicochemical parameters such as culture acidification caused by cell growth or NaDES metabolism.^134^

In general, *in vitro* results agree on the higher toxicity of organic acid-based NaDES. Additionally, Radošević *et al.*^130^ supplemented their findings on microorganisms with cytotoxicity studies on human cancer cell lines. Their study confirmed the significant impact of pH on toxicity, consistent with previous conclusions. However, these findings must be validated by *in vivo* studies, as *in vitro* toxicity results do not always correlate with *in vivo* outcomes. For instance, Mbous *et al.*^135^ compared the cytotoxicity of a DES based on *N*,*N*-diethylethanol ammonium chloride and triethylene glycol (1 : 3) with two NaDES based on ChCl and fructose or glucose (2 : 1). *In vitro* tests showed higher EC_50_ values for NaDES than for the DES, while the opposite was observed *in vivo*. The difference in *in vivo* toxicity was attributed to the significant viscosity of the NaDES studied.

Given their properties, restrictions have been placed on the use of certain NaDES components in specific applications. For example, ChCl, one of the most commonly used and studied quaternary ammonium salts, has been added to the list of substances prohibited in cosmetic products, as specified in Annex II of Regulation (EC) no. 1223/2009 of the European Parliament and Council dated November 30, 2009, due to its irritant properties. Betaine has been proposed as an alternative in cosmetics.^136^

Some precautions should also be taken concerning volatile NaDES. These NaDES, mainly composed of monoterpenes such as menthol, thymol and camphor can expose the user to high amount of the substances. Although these substances have low toxicity,^107^ some acute and chronic toxicity have been reported after exposition of high quantities.^137–139^

Further research into NaDES could help overcome current limitations and expand their range of applications. The non-volatile nature of most NaDES is not always a drawback; in fact, it ensures lower toxicity and can be addressed by rethinking the process. On the other hand, reducing the viscosity of NaDES offers practical advantages and is crucial, as high viscosity can contribute to toxicity. Although some NaDES have been reported to have toxicity, it remains lower than that of traditionally used volatile organic solvents. However, for NaDES to be widely adopted in the pharmaceutical industry, it is essential to gather more comprehensive data on their health and environmental toxicity.

## Conclusions

NaDES offer several advantages, including stability, ease of preparation, biodegradability, broad polarity range, generally low cost, and low toxicity. Their application not only enables the replacement of environmentally or health-hazardous compounds, but also allows taking advantage of their multiple facets. Measurement of green metrics generally shows equivalence or superiority to conventional methods.

NaDES have gained increasing attention in the pharmaceutical industry. In synthesis, NaDES have multiple roles as solvent, catalyst and reagent and are often reused over several reaction cycles. In extraction, as well as generating good yields, they can be used in polar/apolar biphasic systems, in ready-to-use extracts or as pre-treatment solvents. As excipients, NaDES promote the solubility, stability and oral or cutaneous bioavailability of various active ingredients, from small molecules to proteins or DNA. Some NaDES compounds are also API, termed TheDES, or are used as eutectic partners of an API to facilitate its formulation. NaDES are an excellent tool in nanotechnology for monitoring particle formation and ensuring stabilisation. In biotechnology, they serve as a unique medium, suitable for both solubilising organic substrates and stabilising enzymes. These numerous applications suggest promising developments in the coming years.

## Perspectives

This review highlights key areas for future research to improve the use of NaDES in pharmaceutics. These include addressing challenges such as high viscosity and developing more efficient methods for recovery and recycling, which would facilitate the scaling-up of NaDES processes. While large-scale use is already feasible with technical adaptations, refining predictive models of their behaviour is essential for better understanding and implementing NaDES at scale. Additionally, further investigation into their toxicity is needed to support the broader adoption of NaDES as solvents and excipients.

To expand the range of NaDES applications, it is essential to explore other molecules with eutectic properties and varied polarities. One approach would be to revisit their physiological roles in living organisms, particularly in biosynthesis, biomolecule storage and transport, and stress resistance—especially under water stress. In this context, extremophile plants, which have evolved unique adaptations to survive extreme conditions such as intense cold, heat, drought, desiccation, and salinity, could be valuable sources for discovering new eutectic mixtures.^140^

## Conflicts of interest

There are no conflicts to declare.

## Supplementary Material

GC-027-D4GC06386D-s001

## Data Availability

No primary research results, software or code have been included and no new data were generated or analysed as part of this review.
